# Patient Selection and Evidence for Intervention in Deep Vein Thrombosis and Pulmonary Embolism

**DOI:** 10.3390/jcm15218212

**Published:** 2026-10-22

**Authors:** Cezary Maćkowski, Zbigniew Krasiński, Michał-Goran Stanišić, Łukasz Kruszyna, Szymon Salamaga

**Affiliations:** Department of General and Vascular Surgery, University Clinical Hospital in Poznan, Poznan University of Medical Sciences, ul. Długa 1/2, 61-848 Poznan, Poland; zkrasinski@ump.edu.pl (Z.K.); mstanisic@ump.edu.pl (M.-G.S.); lkruszyna@ump.edu.pl (Ł.K.); ssalamaga@ump.edu.pl (S.S.)

**Keywords:** deep vein thrombosis, pulmonary embolism, anticoagulation, catheter-directed thrombolysis, mechanical thrombectomy, post-thrombotic syndrome, patient selection

## Abstract

Intervention for deep vein thrombosis (DVT) and pulmonary embolism (PE) should address a clinical problem that anticoagulation alone may not resolve. This narrative review separates whether to intervene, which procedure to choose, and how to maintain benefit. The review covers publications through 10 September 2026, with a later STORM-PE follow-up report identified separately. Anticoagulation remains foundational. In acute iliofemoral DVT, selected patients may gain earlier symptom relief or less severe post-thrombotic disease, but trials do not support routine thrombus removal. In established post-thrombotic obstruction, C-TRACT showed better six-month clinical severity and quality of life with stenting and enhanced antithrombotic treatment, alongside more bleeding. In selected stable PE, HI-PEITHO reduced a seven-day clinical composite from 10.3% to 4.0%, mainly through fewer episodes of decompensation; a survival benefit remains unproven. Procedure comparisons and imaging trials answer narrower questions than benefit over medical care. Limb-threatening DVT and unstable PE require urgent rescue assessment. A five-scenario table and clinical examples connect this evidence to symptoms, anatomy, recovery goals, bleeding risk, and the feasibility of continuing treatment and follow-up.

## 1. Introduction

Deep vein thrombosis (DVT) and pulmonary embolism (PE) remain a substantial clinical burden. Recent hospital-based PE data illustrate its scale. A nationwide study identified 200,112 patients with PE in 5101 Chinese hospitals in 2021, with an estimated population incidence of 14.2 per 100,000. The 14,123 recorded in-hospital deaths represented a crude case fatality of 7.1%, calculated from the reported counts, and a population death rate of 1.0 per 100,000 [1]. In Poland, PE hospitalization rates were 49 per 100,000 in 2018 and 62 per 100,000 in 2023 (P for trend = 0.059), with a mean in-hospital case fatality of 13% [2]. Differences in case ascertainment, coding, observation periods, and healthcare systems limit direct comparisons.

Treatment options have also expanded. Among US Medicare fee-for-service beneficiaries hospitalized with PE, the proportion receiving catheter-based intervention increased from 5.92% in the second quarter of 2018 to 14.1% in the third quarter of 2023, largely through greater use of mechanical thrombectomy [3]. Increased use makes patient selection more consequential. Restored flow or rapid improvement in imaging may be readily apparent after a procedure, while its effect on survival, disability, or quality of life is harder to establish.

DVT and PE are manifestations of venous thromboembolism (VTE), but the purpose of additional intervention differs. In DVT, clinicians may seek to save a threatened limb, relieve severe acute symptoms, or improve persistent post-thrombotic disability. In PE, the immediate concern is right ventricular failure and circulatory deterioration. Anticoagulation limits propagation and recurrent embolism in both conditions, while these different treatment goals determine which patients and outcomes are relevant to the intervention decision [4,5,6].

We organize this review around three questions that arise in clinical sequence. First, is an invasive strategy likely to improve outcomes beyond anticoagulation and supportive care? If intervention is justified, which procedure best fits the patient’s condition and bleeding risk? Finally, what medication and follow-up are needed to sustain the benefit? This framework helps avoid a common interpretive error: a trial comparing two procedures cannot determine whether either should be added to medical care, and a trial of medication after stenting cannot determine who should receive a stent.

**Figure 1:**
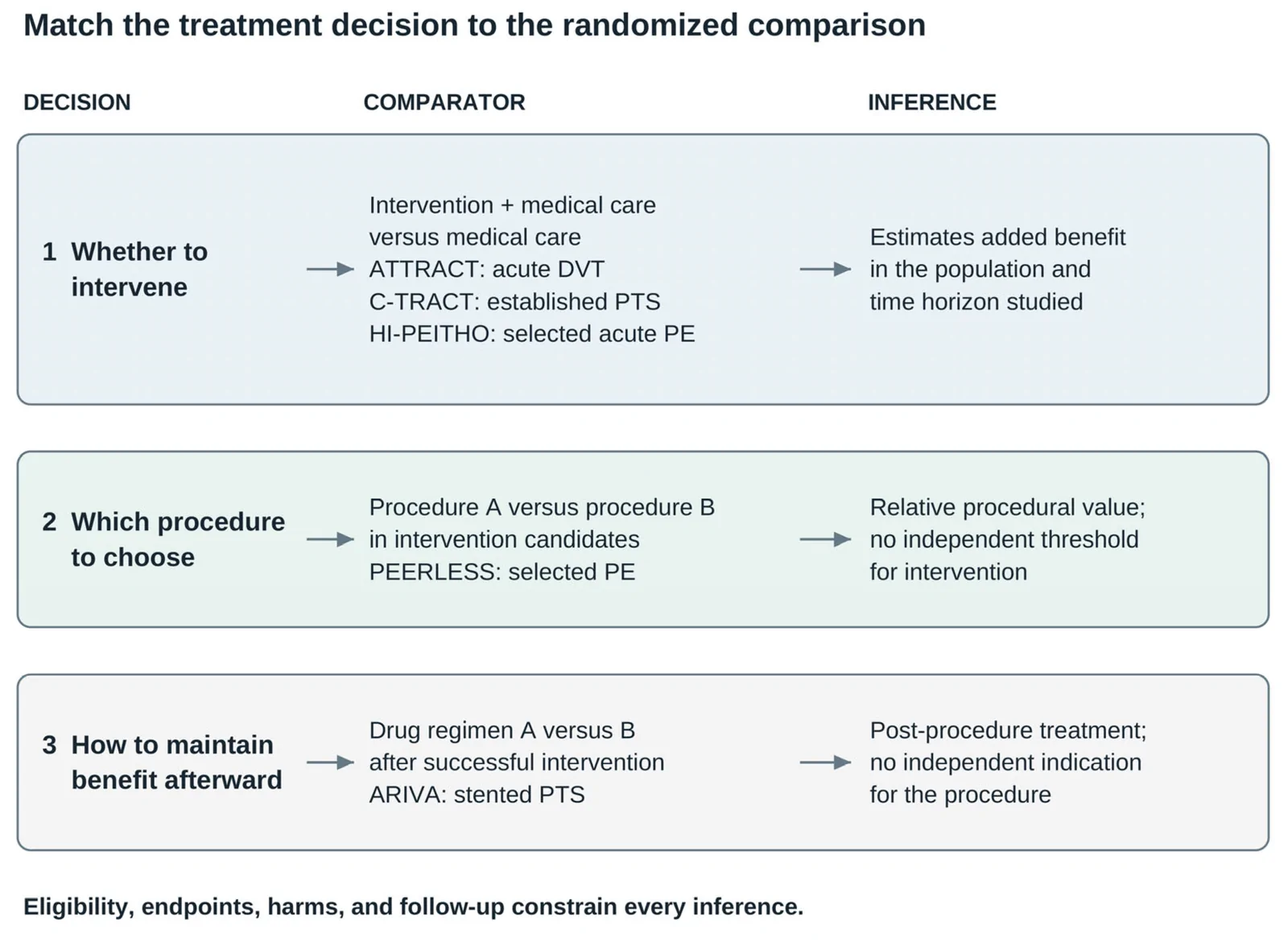
Three treatment decisions and the comparisons needed to answer them. ATTRACT, C-TRACT, and HI-PEITHO evaluate adding an intervention strategy to medical care; PEERLESS compares procedures; ARIVA compares medication after stenting [9,10,11,12,13]. The examples illustrate study questions, not uniformly positive results. DVT, deep vein thrombosis; PE, pulmonary embolism; PTS, post-thrombotic syndrome.

Existing reviews compare international recommendations for acute DVT, and the American Heart Association (AHA) open vein statement connects venous injury with sustained multidisciplinary care [7,8]. We build on that work by bringing acute thrombus removal, reconstruction for established post-thrombotic syndrome (PTS), and pulmonary reperfusion into one clinical argument while respecting their different evidence bases. Figure 1 shows the three treatment decisions.

The main review concerns intervention for established DVT and PE in adults. Caval and upper-extremity thrombosis, pregnancy, cancer, and paradoxical embolism are considered when they alter selection or subsequent treatment. Primary surgical and medical thromboprophylaxis lies outside this central clinical question; a concise background summary is retained in Supplementary Document S1 (Section S8 and Table S6). Pediatric, superficial, cerebral, and splanchnic thrombosis are not comprehensively reviewed.

## 2. Review Approach

To synthesize current evidence for this critical narrative review, targeted literature searches were conducted across four domains: acute DVT thrombus removal, chronic venous reconstruction, PE reperfusion, and antithrombotic medication after intervention. PubMed searches were supplemented by society guidelines, reference lists, publisher reports, public repositories, and ClinicalTrials.gov records. The final reproducible evidence search was conducted on 13 September 2026 and covered publications through 10 September 2026. Targeted verification of individual trial reports and registry records continued through 14 October 2026. The STORM-PE 90-day follow-up, published on 5 October 2026, was included as an explicit exception to the publication cutoff.

Four structured PubMed search strategies returned 1002 unique records across these domains (Supplementary Document S1, Table S1). Separate targeted checks verified individual trial reports, eligibility criteria, outcome populations, guideline grades, and registry status. Supplementary Document S1 records the full search strategies, the dates and scope of these verification checks, and source-access boundaries.

We prioritized randomized comparisons, relevant anatomical and follow-up analyses, guidelines, and prospective registries. Extraction covered population, intervention, comparator, analysis sample, outcomes, follow-up, and harms. Qualitative appraisal considered missing data, masking, co-interventions, multiplicity, applicability, and sponsor roles (Supplementary Document S1, Table S2). Source discrepancies and their treatment are described in Supplementary Document S1; estimates that could not support a reliable numerical interpretation were omitted.

Retrieval was predominantly English-language and non-exhaustive. There was no registered systematic-review protocol, independent duplicate screening, formal risk-of-bias score, or pooled analysis. Guideline grades retain the issuing society’s meaning.

Codex (OpenAI; model: GPT-5.6) and Claude (Anthropic; model: Claude Opus 5.0) assisted with synthesis, drafting, and language editing. Codex also supported retrieval, extraction, comparison, and figure code, as disclosed in the Acknowledgments. Artificial intelligence (AI) assistance was not an independent reviewer or a source of clinical evidence.

## 3. Interpreting Intervention Trials

A trial is most useful when its comparison matches the decision facing the patient. Medical-control trials ask whether an intervention strategy adds benefit. Procedure comparisons help choose a technique once the indication has been established. Post-procedure drug trials address subsequent care. We keep these questions separate throughout the review because favorable results at one stage do not answer the preceding question.

The next step is to identify what improved. Reduced venous obstruction, better right ventricular imaging, fewer episodes of decompensation, less PTS, and improved quality of life are different outcomes. A technical or imaging endpoint can support a mechanism without establishing durable clinical benefit. Composite endpoints also need their components to be examined: fewer episodes of deterioration may be clinically valuable even when mortality is unchanged but should be described as such. Numerical quality-of-life changes require the same scoring method before they can be compared [14].

Uncertainty should then be judged within the study design. A nonsignificant superiority test does not establish equivalence. Noninferiority requires the prespecified margin and analysis to be satisfied, and secondary findings must be read within the testing hierarchy. Follow-up percentages need an explicit population and time point, particularly when outcomes are available for only some participants or are measured per limb. Safety estimates require their own denominators and definitions.

Finally, a poor prognosis is not itself evidence of greater treatment benefit [15]. Severe symptoms may arise from reversible obstruction, established venous injury, or another illness. An exploratory ATTRACT analysis suggested variation in benefit with presenting severity and previous DVT but has not validated an eligibility threshold [16]. Similarly, imaging thresholds derived only in stented patients predict improvement after treatment rather than benefit over medical care [17]. When an applicable comparative effect is established, higher baseline risk may still imply greater absolute benefit. This distinction allows risk, procedural harm, and patient priorities to inform selection without turning a prognostic marker into an automatic indication.

## 4. Foundations of Care

Anticoagulation changed VTE treatment by limiting extension and recurrent embolism without requiring anatomical extraction. Low-molecular-weight heparin enabled outpatient treatment for suitable patients, and direct oral anticoagulants simplified care further [18,19,20,21,22,23,24]. Modern intervention builds on that foundation. Its additional purpose is to relieve a specific threat or disability that persists despite appropriate medical treatment. Selected historical milestones are summarized in Table S3.

Before considering intervention, imaging should define the most proximal affected segment and distinguish acute thrombosis from chronic damage. Clinical probability, D-dimer within an appropriate diagnostic pathway, and compression ultrasonography guide the initial assessment. Continued suspicion after negative proximal imaging warrants reassessment within that pathway [25]. Suspected iliac or caval involvement may require cross-sectional imaging. Iliofemoral DVT includes iliac and/or common femoral thrombosis, with or without distal extension; isolated femoropopliteal disease excludes both segments [4,26]. This distinction is essential when applying trial results.

For most eligible adults with proximal DVT or PE, direct oral anticoagulants are preferred to vitamin K antagonists. Drug choice depends on kidney and liver function, interactions, absorption, adherence, and access. Apixaban and rivaroxaban use intensified initial oral regimens, whereas dabigatran and edoxaban require preceding parenteral anticoagulation [5,6]. High-risk thrombotic antiphospholipid syndrome generally favors a vitamin K antagonist. Obesity alone does not exclude standard-dose apixaban or rivaroxaban, although bariatric surgery and other absorption concerns require separate assessment [27,28].

Initial treatment and extended prevention answer different clinical questions. A resolved major transient provoking factor generally supports stopping anticoagulation after three months, whereas unprovoked or persistent-risk VTE often justifies extended therapy with periodic reassessment of bleeding risk and patient preferences [5,6,29]. Reduced-dose direct oral anticoagulants are an option for selected patients during extended prevention [30,31,32]. Two trials compared reduced- and full-dose direct oral anticoagulants during extended treatment (Table S5). In RENOVE, which enrolled patients at high risk of recurrence, reduced dosing did not meet the noninferiority criterion for recurrent VTE [33]. In API-CAT, which enrolled patients with active cancer after at least six months of treatment, reduced-dose apixaban was noninferior for recurrent VTE and caused less clinically relevant bleeding [34].

Persistent symptoms should prompt a review of dose, adherence, interactions, recurrent thrombosis, and alternative causes before they are attributed to a lesion requiring intervention. Poor vitamin K antagonist control has been associated with later PTS, reinforcing the importance of well-delivered medical care [35]. After thrombus removal, anticoagulation should continue for at least the duration otherwise indicated for the underlying VTE [4].

PRAIS provides exploratory evidence on medication after successful treatment of acute iliofemoral DVT. It compared rivaroxaban with enoxaparin followed by warfarin, randomizing 72 patients and analyzing 67 [36]. The small sample and few recurrence and bleeding events leave the relative efficacy and safety of these regimens uncertain.

Supportive treatment includes mobilization as tolerated, symptom-directed compression, edema and ulcer care, and clear instructions about worsening leg or respiratory symptoms [4,37]. SOX did not demonstrate prevention of PTS with active versus placebo stockings [38]. IDEAL DVT addressed a different decision: after an initial six-month compression period, individualized discontinuation was noninferior to a fixed 24-month course [39]. Compression can therefore be discussed in relation to symptoms, tolerance, skin condition, and reassessment, with prevention of PTS treated as a separate claim.

The practical question is whether medical care has been appropriate and remains feasible. A technically successful procedure does not remove the need for medication, monitoring, or reassessment. These requirements become part of the selection decision before a patient enters the catheter laboratory.

## 5. Patient Selection and Guideline Context

The first distinction is between rescue and elective treatment. Limb-threatening DVT and hemodynamically unstable PE require urgent specialist assessment. In stable presentations, there is time to establish the severity of symptoms, pre-event function, recovery goals, and the likelihood that the proposed procedure will address the cause of disability. Frailty and limited life expectancy may reduce the value of preventing later morbidity while still allowing treatment for immediate limb salvage or severe symptoms [4,37,40]. Table 1 summarizes the five clinical presentations that recur throughout the review, and five hypothetical clinical examples in Section 6, Section 7 and Section 8 illustrate how these principles apply to individual patient decisions.

Guidelines agree that anticoagulation is foundational and that thrombus removal should not be routine in unselected proximal DVT. Their thresholds for selected intervention differ. The European Society for Vascular Surgery (ESVS) considers early removal in selected symptomatic iliofemoral disease and advises against it for isolated femoral, popliteal, or calf thrombosis [4]. The American Society of Hematology (ASH) and CHEST favor anticoagulation alone for most proximal DVT, while the National Institute for Health and Care Excellence (NICE) describes specific criteria for considering iliofemoral catheter-directed thrombolysis [5,6,52].

The Society of Interventional Radiology (SIR) supports carefully selected catheter-directed or pharmacomechanical treatment but does not recommend mechanical thrombectomy without lytics for most acute iliofemoral cases [53]. The European Society of Vascular Medicine (ESVM) gives more favorable recommendations for catheter treatment in severe iliofemoral or iliocaval disease and, within endovascular treatment, favors mechanical thrombectomy over lytic techniques [54].

These differences reflect publication timing, scope, appraisal methods, and judgments about symptom relief and harm [7]. The 2024 CHEST compendium consolidates earlier recommendations rather than replacing them with a wholly new evidence review [29]. Table S7 provides the detailed guideline comparison, including source-specific grades. A recommendation favoring one technique among intervention candidates should not be read as proof that all anatomically eligible patients benefit from intervention.

Safety assessment must distinguish a contraindication to fibrinolysis from inability to receive therapeutic anticoagulation or tolerate vascular access. Avoiding a lytic drug reduces one source of risk but leaves access-site bleeding, blood loss, vascular injury, and subsequent antithrombotic exposure. Recent surgery, active or intracranial bleeding, kidney dysfunction, and concurrent treatment therefore require assessment for the proposed procedure [4,5,54]. Suspected or confirmed heparin-induced thrombocytopenia requires an appropriate diagnostic pathway and non-heparin treatment when indicated [55].

Patient preferences and aftercare can change the balance. Some patients prioritize earlier mobility; others place greater weight on avoiding bleeding or another hospital admission. Before elective stenting, the team should establish an achievable medication and surveillance plan, addressing practical or financial barriers where possible. The treating service must also be able to manage complications and provide reassessment [8,37,53,54]. With these conditions established, anatomy and disease stage can guide the more specific decisions below.

## 6. Acute Deep Vein Thrombosis

In acute DVT, thrombus extent and the clinical treatment goal determine which evidence applies. The following sections distinguish distal and femoropopliteal disease from iliofemoral symptoms and limb-threatening presentations.

### 6.1. Distal and Femoropopliteal Disease

For isolated calf DVT, the usual decision is between anticoagulation and organized ultrasound surveillance. Severe symptoms, extensive or near-proximal thrombus, cancer, previous VTE, and inability to attend surveillance favor treatment. Lower-risk patients managed without anticoagulation need scheduled reassessment and treatment if extension occurs. CHEST describes serial imaging over two weeks, while ESVS recommends reassessment and whole-leg ultrasound after approximately one week [4,6].

CACTUS did not demonstrate a reduction in extension or VTE with nadroparin in low-risk outpatients and reported more bleeding but stopped after approximately half its planned recruitment [56]. RIDTS addressed treatment duration after an uneventful initial six-week rivaroxaban course. Six additional weeks reduced two-year recurrent VTE from 19% to 11%, mainly through fewer distal recurrences [57]. Together, these trials support attention to baseline risk and duration rather than routine intervention. The 2024 International Consensus Statement recommends three months of treatment for symptomatic calf DVT, whereas the earlier European Society of Cardiology (ESC) consensus allows surveillance in selected low-risk patients [30,58].

Anticoagulation also remains standard for isolated femoropopliteal DVT. Extensive thrombus on imaging does not, by itself, justify early removal in this group [4]. Common femoral involvement should be classified as iliofemoral disease because that changes which evidence and recommendations apply.

### 6.2. Selecting Patients with Iliofemoral Disease

Early thrombus removal is most reasonably discussed when recent iliofemoral DVT causes substantial pain, swelling, or loss of mobility in a patient with good pre-event function and low bleeding risk. The anticipated gain should be specific, such as earlier walking or relief of disabling symptoms, and weighed against bleeding and the possibility of further procedures [40,53]. NICE considers catheter-directed thrombolysis (CDT) when symptoms have lasted less than 14 days, functional status is good, expected survival is at least one year, and bleeding risk is low [52]. Trial eligibility windows differed: CaVenT enrolled patients within 21 days of symptom onset and ATTRACT within 14 days [9,41].

Clinical example 1 (Table 1, row 1). A previously active 42-year-old woman has four days of painful left-leg swelling and thrombosis of the external iliac and common femoral veins. Walking remains limited to a few meters despite therapeutic anticoagulation. She has no limb threat or identified major bleeding risk and prioritizes early return to work. This presentation supports discussing early removal alongside medical care. Counseling should distinguish possible earlier mobility and reduced PTS severity from reliable prevention of long-term disability.

Counseling should also cover venous injury, recurrent thrombosis, possible treatment of a residual outflow lesion, and continued anticoagulation. A patient whose main priority is avoiding another invasive treatment may reasonably choose medical care. Conversely, substantial acute disability can make early recovery more valuable, provided the patient understands the limits of the evidence.

### 6.3. What the Randomized Trials Contribute

PTS in these trials is commonly assessed with the Villalta scale, which sums five symptoms and six clinical signs, each scored from 0 to 3. Scores of 5–9 indicate mild PTS, 10–14 moderate PTS, and ≥15 or a venous ulcer severe PTS. The timing and requirement for repeated abnormal assessments differ between studies, so a shared scale does not make their PTS endpoints identical [9,10,41,43].

CaVenT provides evidence that additional CDT can reduce PTS at follow-up in a selected high proximal DVT population. Among 209 randomized patients, PTS at two years was present in 41.1% of assessed intervention patients and 55.6% of assessed controls. The difference remained at five years, with an absolute reduction of 28 percentage points [41,42]. The result concerns PTS status at the assessment visit. Incomplete follow-up and open-label treatment limit interpretation, and improved quality of life or less severe PTS was not demonstrated. We therefore regard CaVenT as support for a selective discussion of thrombus removal, with the outcome and expected benefit made explicit.

ATTRACT tested pharmacomechanical catheter-directed thrombolysis (PCDT) in a larger proximal DVT population. It did not reduce the primary outcome of any PTS and increased early major bleeding [9]. In its prespecified iliofemoral analysis, early symptoms and measures of PTS severity favored PCDT, while any-PTS frequency remained similar [26]. These findings make symptom relief and severity relevant to counseling, rather than supporting routine intervention to prevent all PTS. The overall trial’s moderate-to-severe PTS comparison did not meet its prespecified secondary efficacy threshold; the separate severity-score findings remain informative.

CAVA likewise did not demonstrate a reduction in its one-year primary PTS outcome with ultrasound-accelerated CDT. Its estimate was imprecise, and longer-term findings varied with the definition of PTS [43,44]. Table 2 presents the main results with their analysis populations. Differences in anatomy, eligibility, procedures, and outcome definitions make the trials complementary sources for selection rather than interchangeable estimates of one universal treatment effect.

Earlier randomized studies included aspiration with frequent adjunctive treatment and the heterogeneous TORPEDO endovenous strategy [60,61]. They provide historical comparative evidence but do not settle the effectiveness of each contemporary mechanical platform. Across the evidence, the practical distinction remains between a selected patient seeking relief from substantial iliofemoral symptoms and an unselected patient undergoing removal because a thrombus is visible.

### 6.4. Limb-Threatening DVT

Phlegmasia with threatened limb viability changes the immediate objective from elective symptom improvement to rescue. Management requires urgent specialist assessment, anticoagulation unless contraindicated, resuscitation, and consideration of venous decompression. Catheter, open, or hybrid treatment depends on anatomy, bleeding risk, urgency, and local expertise. Compartment syndrome must be assessed, with fasciotomy when indicated [4,40].

Clinical example 2 (Table 1, row 2). A 58-year-old man with extensive iliofemoral thrombosis develops rapidly increasing swelling, cyanosis, severe pain, and new sensory loss over several hours. The threatened limb requires immediate vascular assessment, therapeutic anticoagulation when feasible, and urgent restoration of venous outflow. Choice of catheter, surgical, or hybrid treatment depends on bleeding risk and the fastest effective option available. Compartment syndrome must be assessed concurrently [4].

Open thrombectomy remains an option when a catheter approach is unsuitable or unavailable. Historical randomized follow-up involved only 30 returning patients and techniques that included a temporary arteriovenous fistula [62]. It helps explain the development of outflow restoration but offers limited guidance for choosing between modern rescue techniques.

### 6.5. Choosing a Technique and Interpreting Registry Recovery

Once intervention is justified, the choice of technique should reflect thrombus characteristics, outflow anatomy, bleeding risk, and service capability. Infusion CDT and ultrasound-assisted CDT both deliver a fibrinolytic. Pharmacomechanical treatment combines mechanical action with variable drug exposure. Mechanical extraction or aspiration may avoid lysis, while retaining risks from access, blood loss, vascular injury, embolization, and recurrent obstruction. Rheolytic systems also carry concern about hemolysis and kidney injury [63]. Table S8 summarizes the mechanisms and available comparisons.

A randomized comparison of Synchronized Wave Aspiration and Maceration with AngioJet in 290 patients found no significant difference in its primary venographic clearance endpoint [64]. This comparison informs device choice among patients already selected for intervention. Establishing an additional clinical benefit over anticoagulation requires a separate randomized comparison.

CLOUT adds prospective information about recovery after ClotTriever treatment. The registry enrolled 500 patients, with 499 in the published analysis population. One-year results were peer-reviewed; the two-year results used here were presented at the VEITH symposium [45,46,59]. Symptoms, venous flow, and Villalta scores improved among those assessed. However, follow-up was incomplete and there was no medical-control group. Anticoagulation, natural recovery, adjunctive treatment, and the procedure therefore contribute inseparably to the observed course, and the denominators in Table 2 distinguish enrolled patients, completed visits, and assessed limbs.

A propensity-matched comparison included 164 CLOUT thrombectomy patients and 164 patients from the ATTRACT anticoagulation arm. At 12 months, PTS was reported in 17% versus 38% of assessed patients [65]. This association supports further randomized evaluation, but matching cannot eliminate differences in patient selection, treatment era, or outcome assessment between the studies. Other observational comparisons are also limited by baseline imbalance and nonstandard PTS definitions (Supplementary Document S1, Section S4.2).

DEFIANCE was designed to address the remaining comparison of a contemporary ClotTriever-based strategy with anticoagulation in symptomatic iliofemoral DVT [66]. Its hierarchical outcome includes treatment failure or escalation followed by PTS severity [67]. Component-specific results and durable function will be needed to translate that comparison into a clearer eligibility discussion.

### 6.6. Caval Thrombosis and Filters

Caval thrombosis requires assessment of the cause as well as the extent of obstruction. Propagating DVT, an indwelling filter, external compression, tumor involvement, and longstanding scar lead to different treatment plans. Anticoagulation remains central where appropriate. Severe symptomatic iliocaval obstruction, organ outflow compromise, or limb threat may justify reconstruction in an experienced center, with attention to bilateral inflow and the underlying disease [4,37].

PREPIC showed a trade-off between fewer pulmonary emboli and more DVT without a survival benefit, while PREPIC2 found no reduction in recurrent PE from adding a retrievable filter to anticoagulation [68,69,70]. These findings support anticoagulation without routine filter placement when therapeutic anticoagulation can be given. A temporary filter may be considered when acute VTE coexists with a contraindication to anticoagulation, accompanied by a retrieval and reassessment plan [5]. FILTER-PEVI examined filters during endovenous intervention in a small randomized trial [71]. Its applicability to contemporary thrombectomy remains uncertain.

### 6.7. Upper-Extremity and Thoracic Outlet Disease

Catheter-associated upper-extremity DVT differs from effort thrombosis associated with thoracic outlet compression. Guidance supports anticoagulation and permits selected early removal for extensive, severely symptomatic disease in suitable patients [72]. A necessary, functioning, noninfected catheter does not invariably require removal. Severe superior vena cava obstruction may need endovascular relief, particularly when symptoms demand prompt treatment, while device- or cancer-related causes must also be addressed.

With convincing venous thoracic outlet compression, decompression and possible first-rib resection become part of the discussion because thrombus removal alone leaves the mechanical cause. The timing of removal, decompression, and adjunctive venoplasty is individualized. Stenting across the thoracic outlet before surgical decompression risks compression-related stent failure [4,72]. These cases illustrate why selecting a procedure requires more than applying lower-limb DVT trial results to another venous territory.

## 7. Established Post-Thrombotic Obstruction

### 7.1. Establishing a Reconstructive Indication

Once post-thrombotic obstruction is established, the treatment objective changes. The question is whether reconstruction can improve disability that persists despite conservative care. Assessment should relate venous claudication, edema, skin changes, or ulceration to obstruction, inflow, reflux, and competing causes of limitation [37]. An incidental iliac compression or the label May-Thurner anatomy is insufficient on its own. Nonthrombotic compression, a residual lesion after acute thrombus removal, and chronic post-thrombotic obstruction have different evidence bases [73] (Supplementary Document S1, Section S12.2).

ESVS recommends considering endovascular treatment for iliac outflow obstruction with severe symptoms or signs, and advises against intervention without severe symptoms [47]. The Society for Cardiovascular Angiography and Interventions (SCAI) conditionally favors iliocaval venoplasty or stenting plus conservative care for symptomatic chronic obstruction, with low certainty in its pre-C-TRACT evidence assessment [48]. Adequate inflow and a feasible antithrombotic and surveillance plan remain part of the indication.

Clinical example 3 (Table 1, row 3). A 55-year-old man remains unable to walk 200 m without venous claudication 18 months after iliofemoral DVT. He has persistent edema, skin changes, and a Venous Clinical Severity Score (VCSS) of 11 despite appropriate medical care. Imaging shows post-thrombotic iliac obstruction with adequate common femoral inflow. These concordant clinical and anatomical findings justify discussing reconstruction and the medication and surveillance it requires. The same narrowing in an asymptomatic patient would provide no comparable clinical reason to intervene.

### 7.2. Reconstruction and Medication After Stenting

Earlier randomized studies by Rossi and Shekarchian reported improvements after stenting but mixed post-thrombotic and nonthrombotic disease and had limited sample sizes [74,75]. C-TRACT directly addressed established moderate or severe PTS with iliac obstruction. Eligibility required substantial activity limitation with VCSS ≥ 8, Villalta ≥ 10, or an open venous ulcer, together with iliac obstruction and adequate inflow. It randomized 225 patients to standard care or a strategy adding stenting and enhanced antithrombotic treatment; 224 entered the modified intention-to-treat analysis [10].

At six months, the C-TRACT strategy reduced clinical severity and improved disease-specific quality of life compared with standard care. The adjusted between-group difference in VCSS was −2.0 points (95% CI −3.2 to −0.8; lower scores indicate less severe disease). The intrinsic VEINES-QoL score was 14.5 points higher (95% CI 9.5–19.4; scale 0–100, higher scores indicate better quality of life). A minimal clinically important difference for VCSS has not been formally established, and quality of life was reported by patients who knew their treatment assignment [10].

Any bleeding occurred in 13/112 versus 4/112 patients. Major bleeding in the intervention arm occurred more than 90 days after the procedure, and most bleeding events occurred months later. Therapeutic anticoagulation and aspirin were recommended for at least six months unless contraindicated. The later bleeding emphasizes the need to discuss continuing medication and bleeding surveillance when choosing the intervention strategy, although the trial cannot attribute the excess to an individual drug. The result supports the tested treatment package over six months, with longer follow-up needed to assess durability [10]. Table 3 distinguishes any bleeding from major bleeding.

ARIVA addresses the next decision after successful post-thrombotic stenting. Adding aspirin to therapeutic rivaroxaban did not demonstrate superior six-month primary patency compared with rivaroxaban alone [13]. The study stopped early and its estimate was imprecise. No major bleeding occurred, while clinically relevant nonmajor bleeding was numerically more frequent with aspirin. We interpret this as an unresolved question about aspirin addition, rather than proof that the regimens are equivalent or that one regimen suits every stented patient.

Post hoc recovery analyses from ARIVA describe recovery after successful stenting [76]. They complement the medication trial but, without an unstented randomized comparator, cannot establish the benefit of reconstruction.

### 7.3. Imaging and Maintaining the Result

Intravascular ultrasound (IVUS) and venography help define the lesion, inflow, and landing zones. In VIDIO, 48 of 68 stented patients had nonthrombotic lesions and 20 had post-thrombotic disease. An IVUS area-stenosis threshold >54% predicted improvement in the mixed cohort, whereas a diameter threshold >61% concerned the nonthrombotic subgroup [17]. These findings inform imaging interpretation alongside symptoms. Their application to post-thrombotic obstruction is limited by the small subgroup and treatment-selected cohort, and area and diameter thresholds are not interchangeable.

Prospective dedicated-stent cohorts such as VIRTUS, ABRE, and VERNACULAR provide longer-term performance data [77,78,79,80,81]. Outcomes differ between nonthrombotic lesions, acute thrombosis, and post-thrombotic disease, so an overall patency percentage can conceal clinically important differences. Table S4 presents patency by disease group and estimation method. These single-arm cohorts describe outcomes in selected populations but cannot establish comparative device performance.

Reconstruction planning should include lesion coverage, vessel dimensions, inflow, and device-specific deployment requirements. Follow-up then needs to address recurrent thrombosis, bleeding, persistent symptoms, and reintervention as well as patency. ESVS describes early duplex surveillance followed by regular review [47]. An open stent with continued disability should prompt reassessment of reflux, residual obstruction, and other causes of limitation. This completes the third treatment decision: maintaining a result that matters to the patient, rather than documenting patency alone.

## 8. Pulmonary Embolism

### 8.1. Clinical Severity and the Purpose of Reperfusion

The pulmonary circulation introduces a different threat: acute right ventricular failure and loss of systemic perfusion. Reperfusion decisions therefore require clinical severity, right ventricular dysfunction, biomarkers, comorbidity, and the course under treatment to be considered alongside clot burden. The ESC framework distinguishes hemodynamically unstable high-risk PE from stable presentations requiring further stratification [50]. The 2026 multisociety guideline from the AHA and American College of Cardiology (ACC) adds categories A–E, subcategories, and a respiratory modifier [51]. Because the two systems define their strata differently, we use each framework’s own terms when discussing its recommendations.

Anticoagulation remains central for stable PE. For unstable patients, systemic thrombolysis, catheter treatment, or surgical embolectomy is selected according to contraindications, urgency, and available expertise. A multidisciplinary PE response team can coordinate this assessment, but the decision still requires a clear treatment objective. In stable patients, the question is whether preventing deterioration justifies the immediate procedural and bleeding risks.

Clinical example 4 (Table 1, row 4). A 63-year-old woman with two days of dyspnea has bilateral proximal PE, a computed tomography (CT) right-to-left ventricular diameter ratio (RV/LV) of 1.3, and elevated troponin. Her systolic pressure remains 105 mmHg for 20 min and her heart rate is 112/min, without hypovolemia, arrhythmia, or sepsis. These findings meet the main HI-PEITHO severity criteria, including systolic pressure ≤ 110 mmHg for at least 15 min and heart rate ≥ 100/min [11]. Anticoagulation, monitored care, and a rescue plan are essential. Catheter treatment can be discussed after full eligibility and bleeding-risk assessment. With normal vital signs and the same CT findings, she would not meet HI-PEITHO’s two-marker distress requirement. She could still meet PRAGUE-26 criteria if her simplified Pulmonary Embolism Severity Index (sPESI) were ≥1 because of another factor, such as a history of cancer, chronic heart failure, or chronic lung disease, provided all other eligibility criteria were satisfied (Table 4).

### 8.2. Clinical Outcomes Compared with Anticoagulation

PEITHO demonstrated the benefit-harm tension of reperfusion in stable intermediate-risk PE. Systemic tenecteplase reduced seven-day death or hemodynamic decompensation, mainly through less decompensation, while increasing serious bleeding and stroke [82]. Its extended follow-up did not demonstrate better long-term survival or symptom recovery [83]. This experience explains why subsequent catheter trials needed to establish clinical benefit with their own regimens and populations.

HI-PEITHO and PRAGUE-26 provide more recent medical-control evidence for catheter-directed thrombolysis. HI-PEITHO enrolled 544 selected stable patients with proximal thrombus, right ventricular dilation, elevated troponin, and at least two prespecified markers of clinical distress. Ultrasound-facilitated CDT reduced the seven-day composite from 10.3% to 4.0%, chiefly through fewer episodes of decompensation [11]. The decompensation endpoint also included sustained National Early Warning Score (NEWS) elevation, as detailed in Supplementary Document S1, Section S4.5. PRAGUE-26 tested conventional alteplase CDT in 558 stable patients with proximal PE, an sPESI ≥ 1, RV/LV ≥ 0.9, and elevated troponin or natriuretic peptide. Unlike HI-PEITHO, it did not require two distress markers. Its corresponding clinical composite occurred in 2 of 280 intervention patients and 19 of 278 controls [49].

These results support discussing catheter-directed reperfusion to reduce early deterioration in appropriately selected, monitored patients. Neither trial established a survival advantage. Bleeding estimates remained imprecise, and the two intracranial hemorrhages in PRAGUE-26 occurred in the intervention group. HI-PEITHO used blinded event adjudication, whereas PRAGUE-26’s committee knew treatment allocation. Differences in eligibility, endpoint definitions, and rescue pathways also prevent ranking the two catheter strategies by their event rates. Table 4 summarizes the clinical results and harms.

### 8.3. Imaging Trials and Procedure Comparisons

ULTIMA and CANARY provided earlier randomized comparisons of catheter treatment with medical care, primarily addressing right ventricular outcomes [87,88]. CANARY stopped early, leaving an imprecise three-month primary estimate. STORM-PE subsequently randomized 100 patients and demonstrated greater RV/LV improvement at 48 h with computer-assisted vacuum thrombectomy plus anticoagulation [84]. Its later 90-day report found longer six-minute walk distance among assessed thrombectomy patients [89]. Testing was completed by 31/47 intervention patients and 34/53 controls. This functional signal complements the early imaging result and supports further study of recovery. Incomplete follow-up and the small sample limit conclusions about durability, survival, and comparative safety.

STRATIFY compared ultrasound-assisted thrombolysis, intravenous low-dose alteplase, and heparin. Pooled thrombolysis improved clot-burden reduction, but ultrasound-assisted treatment did not demonstrate superiority over intravenous delivery [85,86]. SUNSET sPE likewise found no greater thrombus clearance with ultrasound-assisted than standard CDT [90]. These findings help define what individual treatment components may contribute while preserving the distinction between an imaging result and recovery meaningful to patients. Smaller trials, including PRETHA, BETULA, and an earlier pilot, remain too imprecise to establish durable clinical benefit [91,92,93].

Other randomized comparisons help refine procedure choice. OPTALYSE PE compared four ultrasound-facilitated lytic regimens in 101 patients and documented RV/LV improvement within the treatment groups [94]. SPECIAL compared ultrasound-assisted thrombolysis with surgical embolectomy in 27 patients before stopping early. Its 72 h RV/LV analysis was inconclusive for noninferiority [95]. Both trials inform technique research, with their scope limited by imaging endpoints and the absence of an anticoagulation-only group.

PEERLESS answers the second question in our framework. Among patients selected for intervention, its hierarchy of death, intracranial hemorrhage, major bleeding, deterioration or bailout, and intensive care use favored large-bore thrombectomy over CDT through discharge or seven days, whichever came first. The four-component hierarchy excluding intensive care use was inconclusive [12]. Of 599 randomized patients, 550 entered the enrolled and treated analysis population. The result can inform procedure choice in that setting, but there was no anticoagulation-only arm. Procedure-specific monitoring requirements also matter when interpreting intensive care use.

### 8.4. Rescue Treatment and Recovery

Medical treatment for PE includes active monitoring and access to rescue treatment. Reassessment should integrate perfusion, blood pressure, oxygen needs, symptoms, and the trajectory of right ventricular dysfunction. The favorable early composites in HI-PEITHO and PRAGUE-26 were observed within such care pathways. A patient who deteriorates may move outside an elective trial population and require an urgent decision based on the current threat [11,49,50,51].

Clinical example 5 (Table 1, row 5). A 70-year-old man with confirmed PE has persistent systolic pressure of 80 mmHg, confusion, and rising lactate despite initial support. The immediate task is stabilization and rapid reperfusion. Systemic thrombolysis is an option if bleeding risk permits; catheter treatment or surgical embolectomy may be appropriate when lysis is contraindicated or unsuccessful and the required expertise is available. Elective counseling must not delay rescue. In refractory shock, venoarterial extracorporeal support may provide a bridge in an experienced service [50,51].

After reperfusion, anticoagulation and follow-up remain essential. RE-SPIRE compared dabigatran with warfarin after a procedure, but its small population and sparse events do not define a universal drug regimen or duration [96]. At approximately three months, persistent dyspnea or exercise limitation warrants assessment for recurrence, comorbidity, deconditioning, and chronic thromboembolic disease when suspected. Appropriate echocardiography, perfusion imaging, specialist referral, and individualized rehabilitation connect this assessment to recovery; routine surveillance imaging is not recommended for asymptomatic patients [97].

## 9. Special Clinical Populations

The same three treatment decisions apply in special populations, although competing risks and treatment constraints change their balance.

Pregnancy changes anticoagulant choice and procedural planning. Low-molecular-weight heparin is preferred for most pregnancy-associated VTE, with treatment generally continuing for at least six weeks postpartum and at least three months in total [98]. Delivery planning requires coordination with obstetrics and anesthesia. In pregnant patients with suspected PE, the pregnancy-adapted YEARS algorithm can guide the need for chest imaging [99]. For intervention, limb threat or severe cardiopulmonary compromise must be weighed against maternal bleeding, radiation and medication exposure, and gestational timing. Pregnancy alone is not an indication for elective thrombus removal.

In cancer-associated VTE, recurrence and bleeding risk depend on tumor site, mucosal lesions, platelet count, organ function, absorption, and treatment interactions. Guidance supports low-molecular-weight heparin or selected direct oral anticoagulants [100]. Caravaggio, Hokusai VTE Cancer, and SELECT-D illustrate why the drug and cancer population both matter [101,102,103]. API-CAT supports reduced-dose apixaban for extended treatment in its selected population after at least six months, with results summarized in Table S5 [34]. Malignancy-related venous obstruction may also involve external compression or tumor, making the anticipated symptom benefit, prognosis, and feasibility of continuing anticoagulation central to reconstruction.

Paradoxical embolism requires a plausible venous source and right-to-left shunt, together with assessment for other causes of the arterial event. A patent foramen ovale (PFO) alone does not establish causation. Guidance does not support closure solely for coexisting DVT and a PFO; selected PFO-associated stroke with a continuing anticoagulation indication is a separate, low-certainty decision [104]. Closure does not replace anticoagulation required for DVT or PE. Additional attribution and closure evidence is summarized in Supplementary Document S1 (Section S12.2).

These populations emphasize the limits of transferring average trial effects into patients with different competing risks. The treatment goal remains the same: a clinically meaningful improvement that is plausible for the patient’s condition and achievable within the necessary ongoing care.

## 10. Discussion and Conclusions

### 10.1. Applying the Evidence Across the Treatment Course

**Figure 2:**
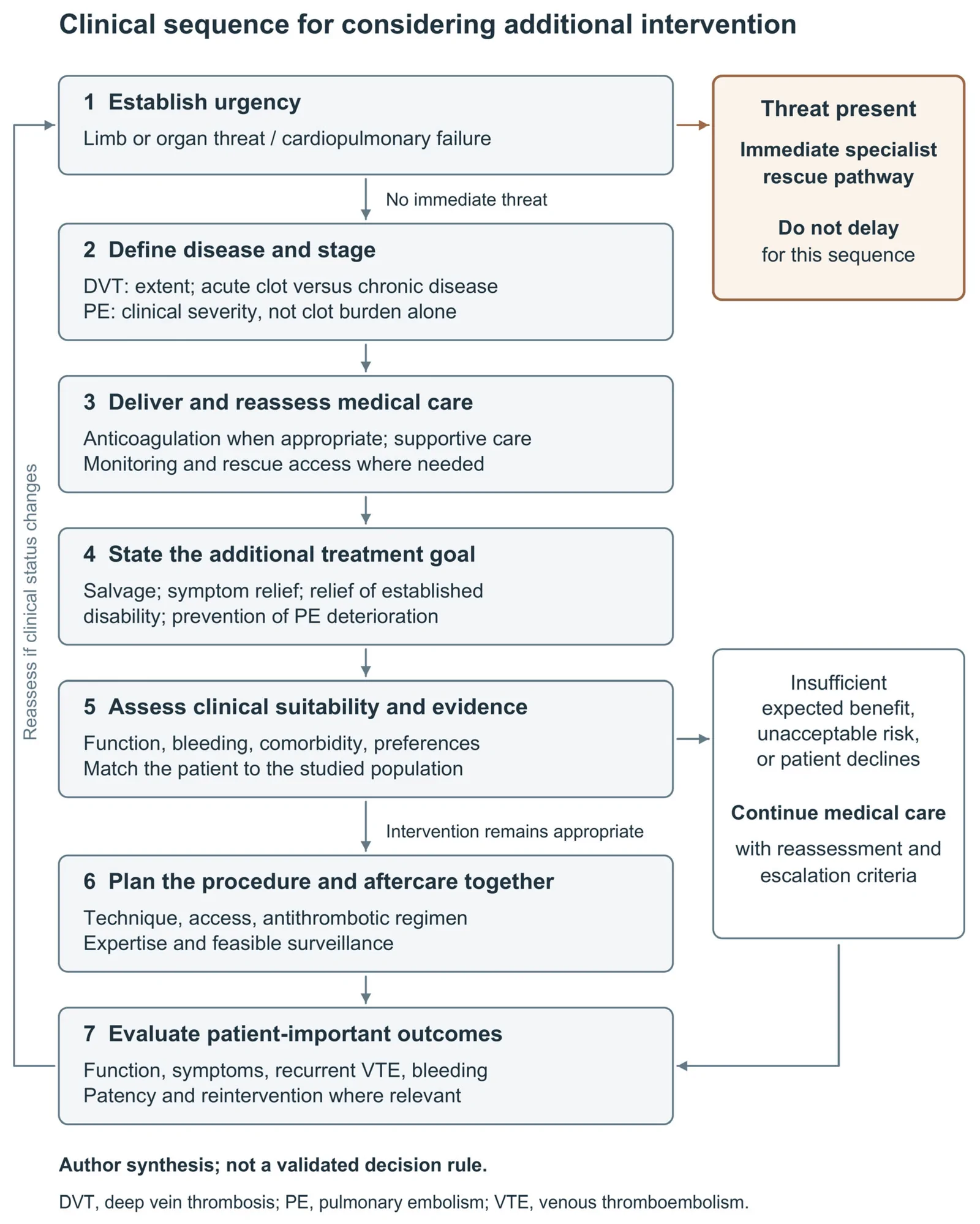
Clinical sequence for considering intervention in DVT or PE. Urgent limb threat or cardiopulmonary failure takes priority. In stable patients, the sequence connects diagnosis, medical treatment, clinical goals, suitability, procedure planning, and reassessment [4,5,37,51,52,54]. Continuing medical care includes monitoring and escalation when needed. VTE, venous thromboembolism.

This review organizes patient selection around three linked decisions: whether intervention adds benefit, which procedure to choose, and how benefit is maintained afterward. Table 1 identifies the clinical presentation, treatment goal, and relevant evidence. Figure 2 then guides the sequence of assessment: identify an immediate threat, provide appropriate medical care, define the additional benefit sought, and plan any procedure together with aftercare. A patient’s presentation may change, for example from stable to unstable PE, requiring renewed assessment of urgency and treatment options.

For elective treatment, the most useful conversation begins with the outcome the patient wants to improve. Early walking after acute iliofemoral DVT, relief of established venous claudication, and avoidance of early deterioration in selected PE are distinct goals. Evidence for one should not be substituted for evidence for another. We would frame counseling around the likely magnitude and timing of that benefit, the important harms, and the demands of subsequent care.

The medical comparator also deserves attention. Appropriate anticoagulation, adherence support, symptom management, and timely reassessment are active treatment. In PE, monitoring and rescue access are part of that strategy. Persistent disability despite well-delivered care can justify reassessing anatomy and goals, while improvement under medical treatment can change the value of an elective procedure. Selection is therefore revisited as the clinical course evolves.

### 10.2. Research Priorities

Contemporary trials should measure durable function and quality of life alongside early technical success. In DVT, PTS occurrence and severity, recurrent thrombosis, bleeding, and reintervention need separate reporting. In PE, prevention of early decompensation should be linked to longer-term exercise capacity, symptoms, and survival. Clear co-intervention protocols, crossover reporting, and outcome-specific denominators would make these results easier to apply.

DEFIANCE addresses a modern mechanical strategy in acute iliofemoral DVT; PEERLESS II compares pulmonary thrombectomy with anticoagulation; PE-TRACT evaluates exercise and functional recovery; and PEITHO-3 evaluates reduced-dose systemic reperfusion [67,105,106,107,108,109]. No primary results reports from PEERLESS II or PEITHO-3 were identified in targeted checks completed on 14 October 2026. Table S9 describes the trial programs; dates and the scope of those checks are specified in Supplementary Document S1, Section S11.

Further work should test selection hypotheses using randomized comparisons across patient groups and report both absolute benefit and harm. Imaging-derived prognosis alone cannot establish who benefits from intervention. After venous stenting, the optimal antithrombotic regimen and duration also remain uncertain. Emerging anticoagulants may alter this balance, but efficacy must be established for the relevant VTE indication (Supplementary Document S1, Section S12.1).

### 10.3. Limitations

This selective narrative review used predominantly English-language sources and did not include independent duplicate screening, formal risk-of-bias grading, or meta-analysis. Two-year CLOUT outcomes came from a conference presentation [59], and the 90-day STORM-PE report was assessed from its published abstract [89]. Supplementary Document S1 identifies the source versions and access limitations. Related publications from the same trial were treated as overlapping evidence rather than independent cohorts.

Evidence remains limited for several special populations, unusual venous sites, long-term outcomes, and medication after reconstruction. Guidance and registry status can change after the stated retrieval dates. The clinical examples, summary table, and figures illustrate interpretation and discussion; they do not constitute validated prediction rules or replace disease-specific guidance.

### 10.4. Conclusions

Anticoagulation remains the foundation of DVT and PE treatment. Additional intervention should be matched to a defined clinical problem: limb threat, substantial acute iliofemoral symptoms, disabling post-thrombotic obstruction, or selected pulmonary presentations at risk of deterioration. The relevant evidence differs between these settings. Acute-DVT trials support selective symptom and severity discussions, C-TRACT supports a reconstructive and antithrombotic strategy for established disease, and recent catheter-directed PE trials demonstrate fewer early clinical events in selected stable patients. These benefits must be weighed against bleeding and the need for continuing care.

Separating whether to intervene, which procedure to choose, and how to maintain benefit makes that judgment more transparent. Clinical success should ultimately be assessed through survival, function, symptoms, and treatment burden over time.

## Figures and Tables

**Table 1:** Five clinical presentations organized by the decision to intervene, procedure choice, and subsequent care.

Clinical Presentation	Whether to Intervene and Goal	Procedure Choice	Maintaining Benefit	Key Evidence and Guidance
1. Acute iliofemoral DVT without limb threat	Consider disabling recent symptoms, good baseline function, and low bleeding risk. Goal: earlier relief and less severe PTS.	Match catheter thrombolysis or removal to anatomy and risk; device evidence differs.	Continue indicated anticoagulation; reassess symptoms and outflow.	CaVenT, ATTRACT, CAVA [9,26,41,42,43,44]; CLOUT registry [45,46]. ESVS IIa A [4].
2. Limb-threatening DVT	Urgent rescue for threatened viability. Goal: limb salvage.	Catheter, open, or hybrid removal; assess compartment syndrome.	Anticoagulation when feasible; close limb and outflow assessment.	ESVS phlegmasia guidance [4]; limited randomized rescue evidence.
3. Established post-thrombotic obstruction	Consider persistent moderate/severe disability with iliac obstruction, adequate inflow, and feasible aftercare.	Reconstruct a lesion concordant with symptoms.	Antithrombotics, surveillance, symptom/ulcer care, and bleeding review.	C-TRACT [10]; ARIVA for aspirin addition [13]. ESVS: endovascular treatment for severe symptoms/signs, IIa B [47]. SCAI: iliocaval venoplasty/stenting plus conservative care, conditional recommendation with low-certainty evidence [48].
4. Stable intermediate-risk PE	Anticoagulation and monitoring. Consider selected intermediate-high-risk presentations matching trial criteria; goal: less early deterioration.	Assess catheter reperfusion against bleeding risk and clinical course.	Maintain rescue access, anticoagulation, and functional follow-up.	HI-PEITHO, PRAGUE-26 [11,49]. ESC: routine systemic lysis III B [50]. AHA/ACC C2–C3: benefit of CDT or MT over anticoagulation remains unclear (2b C-LD) [51].
5. Hemodynamically unstable PE	Urgent stabilization and reperfusion. Goal: reverse circulatory failure.	Systemic lysis; catheter or surgical rescue according to contraindications and expertise.	Critical care, appropriate anticoagulation, and organ-recovery assessment.	ESC: systemic lysis I B; surgery I C and catheter treatment IIa C when lysis is contraindicated/failed [50].

This table synthesizes the cited evidence. Anticoagulation accompanies intervention unless contraindicated. Routine prevention of any PTS and a survival benefit in stable PE remain unproven. Guideline classes apply to the specified treatment and population; Table S7 gives source-specific recommendations. ESC 2019 and AHA/ACC 2026 guidance preceded publication of HI-PEITHO and PRAGUE-26 and should be read alongside the later trial evidence. ACC, American College of Cardiology; AHA, American Heart Association; CDT, catheter-directed thrombolysis; MT, mechanical thrombectomy; C-LD, limited data; ESC, European Society of Cardiology; ESVS, European Society for Vascular Surgery; SCAI, Society for Cardiovascular Angiography and Interventions; DVT, deep vein thrombosis; PE, pulmonary embolism; PTS, post-thrombotic syndrome. The numbered examples correspond to the five clinical rows.

**Table 2:** Acute DVT intervention evidence with explicit enrollment and outcome populations.

Study and Comparison	Randomized or Enrolled Population and Analysis	Principal Outcomes at Stated Follow-Up	Clinical Interpretation
CaVenT [41,42] CDT + standard care vs. standard care	209 randomized (101 vs. 108). Assessed: 189 at 2 years (90 vs. 99); 176 at 5 years (87 vs. 89).	PTS at 2 years: 37/90 (41.1%) vs. 55/99 (55.6%); ARR 14.4 pp (95% CI 0.2–27.9). At 5 years: 37/87 (42.5%) vs. 63/89 (70.8%); ARR 28 pp (95% CI 14–42). Three acute major bleeds with CDT.	Less PTS at assessment; no demonstrated quality-of-life gain.
ATTRACT [9] PCDT + anticoagulation vs. anticoagulation	692 randomized; 691 analyzed (336 vs. 355). Cumulative PTS occurrence over 6–24 months.	Any PTS: 157/336 (47%) vs. 171/355 (48%). Moderate/severe PTS: 18% vs. 24% (*p* = 0.04; secondary threshold *p* ≤ 0.01). 10-day major bleeding: 1.7% vs. 0.3%.	Negative primary PTS outcome; more early bleeding.
ATTRACT iliofemoral analysis [26]	391 in the prespecified anatomical stratum (196 vs. 195); overlaps the parent trial.	Any PTS over 6–24 months: 96/196 (49%) vs. 100/195 (51%). Moderate/severe PTS: 18% vs. 28%. Improved early symptoms and severity measures.	Selective symptom/severity discussion; overlapping trial population.
CAVA [43,44] Ultrasound-accelerated CDT + standard care vs. standard care	184 randomized; 152 in modified ITT analysis (77 vs. 75).	1-year PTS (among 77 vs. 75 mITT patients): 29% vs. 35%; OR 0.75 (95% CI 0.38–1.50). Major bleeding: 5% vs. 0%.	Imprecise primary estimate; definition-sensitive longer follow-up.
CLOUT at 1 year [45,46] Single-arm registry	500 enrolled; 499 analyzed patients (521 limbs). 310 patients completed visits; 285 limbs had Villalta assessment.	PTS: 55/285 limbs (19.3%). Moderate/severe PTS: 25/285 limbs (8.8%).	Recovery among assessed limbs; no medical-control comparison.
CLOUT at 2 years [59] Conference presentation	Same registry. 285 patients completed follow-up; 265 limbs had Villalta assessment.	PTS: 18.5% of 265 limbs. Moderate/severe PTS: 6.4% of 265 limbs.	Descriptive durability with attrition; conference evidence.

Paired results are intervention vs. control. ATTRACT reports cumulative PTS occurrence over 6–24 months; percentages for moderate/severe PTS and major bleeding refer to the respective analyzed populations (336 vs. 355 overall; 196 vs. 195 in the iliofemoral stratum). CaVenT percentages use assessed patients at each follow-up visit rather than cumulative incidence across all randomized participants. CLOUT percentages refer to assessed limbs rather than all enrolled patients (the two-year conference report provided percentages among 265 assessed limbs without exact event counts). Efficacy and safety populations may differ. ARR, absolute risk reduction; CDT, catheter-directed thrombolysis; CI, confidence interval; ITT, intention-to-treat; mITT, modified intention-to-treat; OR, odds ratio; PCDT, pharmacomechanical CDT; pp, percentage points; PTS, post-thrombotic syndrome.

**Table 3:** Established PTS and medication after stenting answer different treatment questions.

Study and Population	Treatment Question and Comparison	Six-Month Result	Bleeding and Interpretation
C-TRACT [10] 225 randomized; 224 modified ITT	Add reconstruction in moderate/severe PTS with iliac obstruction? Stenting + enhanced antithrombotics + standard care vs. standard care.	Adjusted VCSS difference: −2.0 (95% CI −3.2 to −0.8). VEINES-QoL difference: 14.5 (9.5–19.4), intrinsic 0–100 scale; higher is better.	Any bleeding: 13/112 vs. 4/112; adjusted RR 3.22 (1.07–9.69). Major bleeding: 4/112 vs. 1/112. Benefit of the treatment package.
ARIVA [13] 169 randomized; 162 full analysis set; 156 primary efficacy	Add aspirin after successful PTS stenting? Aspirin 100 mg + rivaroxaban 20 mg daily vs. rivaroxaban alone.	Primary patency: 73/77 (94.8%) vs. 73/79 (92.4%). Difference: 2.4 pp (reported 95% CI −13.6 to 18.0).	No major bleeding. CRNMB: 7/85 vs. 2/84 patients. Aspirin superiority not demonstrated; early stopping and imprecision.

C-TRACT bleeding categories overlap and must not be added; its safety *p* value was nominal. ARIVA bleeding uses the 169-patient safety population. CI, confidence interval; CRNMB, clinically relevant nonmajor bleeding; ITT, intention-to-treat; pp, percentage points; PTS, post-thrombotic syndrome; RR, risk ratio; VCSS, Venous Clinical Severity Score; VEINES-QoL, Venous Insufficiency Epidemiological and Economic Study Quality of Life. Detailed appraisal is in Supplementary Document S1; dedicated-stent cohorts are in Table S4.

**Table 4:** PE trials grouped by clinical outcomes, imaging outcomes, and procedure comparison.

Trial and Analysis Population	Randomized Comparison	Primary Result and Time Point	Key Harms
PEITHO [82,83] Clinical outcome 1006 randomized; 1005 reported ITT	Tenecteplase + heparin vs. placebo + heparin	7-day death/decompensation: 13/506 (2.6%) vs. 28/499 (5.6%); OR 0.44 (95% CI 0.23–0.87).	ISTH major bleeding: 58/506 (11.5%) vs. 12/499 (2.4%). All stroke: 2.4% vs. 0.2%.
HI-PEITHO [11] Clinical outcome 544 randomized (273 vs. 271); 542 in the safety population (271 vs. 271). Age 18–80; proximal PE; RV/LV ≥ 1.0; troponin elevation; ≥2 distress markers. *	Ultrasound-facilitated CDT + anticoagulation vs. anticoagulation	7-day PE-related death, cardiorespiratory decompensation/collapse, or symptomatic recurrent PE: 11/273 (4.0%) vs. 28/271 (10.3%); RR 0.39 (95% CI 0.20–0.77).	7-day ISTH major bleeding: 11/271 (4.1%) vs. 6/271 (2.2%); RR 1.8 (95% CI 0.7–4.9). No intracranial hemorrhage occurred in either group through 30 days.
PRAGUE-26 [49] Clinical outcome 558 randomized (280 vs. 278). Age > 18 to ≤80 years; proximal PE < 14 days; sPESI ≥ 1; RV/LV ≥ 0.9; troponin or natriuretic peptide elevation.	Alteplase CDT + anticoagulation vs. anticoagulation	7-day all-cause death, recurrent PE, or decompensation/collapse: 2/280 vs. 19/278; RR 0.10 (95% CI 0.02–0.44).	ISTH major bleeding: 5/280 vs. 7/278; RR 0.71 (0.23–2.21). Intracranial hemorrhage: 2/280 vs. 0/278.
STORM-PE [84] Imaging outcome 100 randomized (47 vs. 53)	Vacuum thrombectomy + anticoagulation vs. anticoagulation	48 h RV/LV reduction: 0.52 vs. 0.24; reported difference 0.27 (95% CI 0.12–0.43).	7-day MAE: 2/47 vs. 4/53. PE-related deaths: 2 vs. 0.
STRATIFY [85,86] Imaging outcome 210 randomized (71 vs. 70 vs. 69)	USAT vs. intravenous alteplase vs. heparin; both lytic arms 20 mg/6 h with heparin	48–96 h rmMS difference: pooled lysis vs. heparin −3.6 (95% CI −5.0 to −2.2); USAT vs. intravenous −0.1 (−1.9 to 1.7).	TIMI major bleeding: 2/71, 2/70, 0/69. 3-month deaths: 2/71, 4/70, 0/69.
PEERLESS [12] Procedure comparison 599 randomized; 550 enrolled/treated	Large-bore thrombectomy vs. CDT; no medical-only arm	Through discharge or day 7, whichever first: primary win ratio 5.01 (95% CI 3.68–6.97). Four-component hierarchy without ICU use: 1.34 (0.78–2.35).	Major bleeding: 19 patients in each arm (6.9% each). No demonstrated mortality or bleeding difference; no equivalence claim.

* HI-PEITHO required ≥2 of: systolic pressure ≤ 110 mmHg for ≥15 min; heart rate ≥ 100/min; respiratory rate > 20/min or hypoxemia. Selected eligibility criteria and key exclusions are summarized in Supplementary Document S1, Section S4.5; complete criteria are provided in the original trial reports and registry records. sPESI, simplified Pulmonary Embolism Severity Index. Pairs follow the listed comparison; STRATIFY triples follow its listed arms. Clinical composites were driven mainly by decompensation and do not establish survival benefit. Safety definitions and populations are not interchangeable; HI-PEITHO uses treated participants, and PRAGUE-26 uses all randomized participants. PRAGUE-26 also reported seven-day major bleeding according to GUSTO criteria: 4/280 versus 6/278. These counts use a different bleeding definition from the ISTH results shown above. GUSTO, Global Use of Strategies to Open Occluded Coronary Arteries; CDT, catheter-directed thrombolysis; CI, confidence interval; ICU, intensive care unit; ISTH, International Society on Thrombosis and Haemostasis; ITT, intention-to-treat; MAE, major adverse event; OR, odds ratio; PE, pulmonary embolism; rmMS, refined modified Miller score; RR, risk ratio; RV/LV, right-to-left ventricular diameter ratio; TIMI, Thrombolysis In Myocardial Infarction; USAT, ultrasound-assisted thrombolysis.

## Data Availability

No original patient-level dataset was generated. Published data were extracted and critically synthesized; source-specific notes are provided in Supplementary Document S1. The underlying published data are available in the cited publications.

## Supplementary Materials

The following supporting information can be downloaded at https://www.mdpi.com/article/10.3390/jcm15218212/s1. Supplementary Document S1, “Supporting Evidence for Intervention in Deep Vein Thrombosis and Pulmonary Embolism”, contains the following tables: Table S1: Reproducible PubMed strategies executed on 13 September 2026 (publication cutoff: 10 September 2026). Table S2: Selected evidence and qualitative appraisal, grouped as acute DVT/compression, chronic reconstruction, PE, medication/prophylaxis, and guidance. Table S3: Selected historical milestones. Table S4: Dedicated venous stent cohorts and estimation methods. Table S5: Anticoagulation and prophylaxis trials. Table S6: Primary surgical and medical thromboprophylaxis: selected settings and regimen considerations. Table S7: Selected guideline recommendations and their original grades. Table S8: Interventional mechanisms and relevant evidence. Table S9: Selected trial programs on 13 September 2026. Section S12, “Additional clinical context”, covers emerging anticoagulants, paradoxical embolism, and nonthrombotic compression. References [110,111,112,113,114,115,116,117,118,119,120,121,122,123,124,125,126,127,128,129,130,131,132,133,134,135,136,137,138] are cited in the Supplementary Materials.

## Abbreviations

ACC	American College of Cardiology
AHA	American Heart Association
AI	artificial intelligence
ARR	absolute risk reduction
ASH	American Society of Hematology
CDT	catheter-directed thrombolysis
CI	confidence interval
CRNMB	clinically relevant nonmajor bleeding
CT	computed tomography
DVT	deep vein thrombosis
ESC	European Society of Cardiology
ESVM	European Society of Vascular Medicine
ESVS	European Society for Vascular Surgery
GUSTO	Global Use of Strategies to Open Occluded Coronary Arteries
ICU	intensive care unit
ISTH	International Society on Thrombosis and Haemostasis
ITT	intention-to-treat
IVUS	intravascular ultrasound
MAE	major adverse event
mITT	modified intention-to-treat
MT	mechanical thrombectomy
NEWS	National Early Warning Score
NICE	National Institute for Health and Care Excellence
OR	odds ratio
PCDT	pharmacomechanical catheter-directed thrombolysis
PE	pulmonary embolism
PFO	patent foramen ovale
PTS	post-thrombotic syndrome
rmMS	refined modified Miller score
RR	risk ratio
RV/LV	right-to-left ventricular diameter ratio
SCAI	Society for Cardiovascular Angiography and Interventions
SIR	Society of Interventional Radiology
sPESI	simplified Pulmonary Embolism Severity Index
TIMI	Thrombolysis In Myocardial Infarction
USAT	ultrasound-assisted thrombolysis
VCSS	Venous Clinical Severity Score
VEINES-QoL	Venous Insufficiency Epidemiological and Economic Study Quality of Life
VTE	venous thromboembolism
