# Supplementary material for: Patient Selection and Evidence for Intervention in Deep Vein Thrombosis and Pulmonary Embolism
Source: J Clin Med. 2026 Oct 22;15(21):8212. doi: 10.3390/jcm15218212 (PMC13763735; doi:10.3390/jcm15218212)
Supplement: Supplementary file 1 [file jcm-15-08212-s001.zip › jcm-4606473-supplementary.pdf]

Supplementary Document S1

Supporting Evidence for Intervention in Deep Vein Thrombosis and Pulmonary Embolism

This supplement documents retrieval methods, study appraisal, outcome populations, and clinical background for the accompanying narrative review. Reference numbers refer to the main manuscript. Tables S1–S9 cover search strategies, appraisal, historical milestones, stent cohorts, medication trials, surgical prophylaxis, guidelines, techniques, and trial registries.

Additional abbreviations: NYHA, New York Heart Association; CIVIQ-20, 20-item Chronic Venous Insufficiency Questionnaire; GUSTO, Global Use of Strategies to Open Occluded Coronary Arteries; AUC, area under the curve; CTEPH, chronic thromboembolic pulmonary hypertension; NIH, National Institutes of Health.

S1. Review design

This critical narrative review examined primary trial publications, society guidelines, and public clinical trial registries across acute DVT thrombus removal, chronic venous reconstruction, PE reperfusion, and post-intervention antithrombotic therapy. Selection prioritized randomized comparisons, relevant subgroup and follow-up reports, contemporary guidelines, and prospective registries. Historical reports explain changes in treatment goals. Retrieval was predominantly English-language and non-exhaustive, with no registered systematic-review protocol, duplicate independent screening, formal risk-of-bias score, or meta-analysis. AI tool use and author oversight are described in Section 2 and the Acknowledgments of the main manuscript.

S2. Retrieval and bibliographic verification

The final reproducible PubMed evidence search was executed on 13 September 2026 (yielding identical record counts to an earlier run on 11 September 2026), with a publication cutoff of 10 September 2026. The STORM-PE 90-day follow-up report, published on 5 October 2026, was added as an explicit exception [89]. The initial search phase used PubMed topic, trial-name, title, and identifier searches, supplemented by society guideline websites, reference lists, publisher reports, and public repositories. The seven-trial ClinicalTrials.gov registry snapshot (Table S9) was obtained on 13 September 2026, and targeted verification of individual publications, eligibility criteria, guideline grades, and PEERLESS II/PEITHO-3 result availability continued through 14 October 2026. These checks did not update the remaining five registry entries or change the publication cutoff of 10 September 2026.

Table S1 lists the four reproducible PubMed strategies that constitute the final evidence search (13 September 2026). Earlier exploratory searches used different queries; their record counts are not combined with the Table S1 totals. All later activities were targeted verification checks of individual reports, registry records, and guideline grades, not additional evidence searches.

Table S1. Reproducible PubMed strategies executed on 13 September 2026 (publication cutoff: 10 September 2026).

Domain	Exact PubMed query	Count	Selected benchmark reports retrieved
Acute DVT removal	("Venous Thrombosis"[Mesh] OR "deep vein thrombosis"[tiab] OR "deep venous thrombosis"[tiab] OR iliofemoral[tiab]) AND ("Thrombolytic Therapy"[Mesh] OR "Thrombectomy"[Mesh] OR "catheter-directed"[tiab] OR thrombectomy[tiab] OR pharmacomechanical[tiab]) AND ("Randomized Controlled Trial"[pt] OR randomized[tiab] OR randomised[tiab] OR registry[tiab]) NOT ("Pulmonary Embolism"[Majr] OR "Stroke"[Majr] OR "Cerebral Venous Thrombosis"[tiab])	357	11/11: ATTRACT main/anatomical; CaVenT 2/5 years; CAVA primary/follow-up; CLOUT 6 months/1 year; aspiration RCT; TORPEDO; SWAM [9, 26, 41, 42, 43, 44, 45, 46, 60, 61, 64].
Venous stenting	("Postthrombotic Syndrome"[Mesh] OR "Iliac Vein"[Mesh] OR "post-thrombotic"[tiab] OR postthrombotic[tiab] OR "iliac vein"[tiab] OR "iliofemoral vein"[tiab] OR "venous outflow obstruction"[tiab]) AND ("Stents"[Mesh] OR stent[tiab] OR stenting[tiab] OR recanalization[tiab]) AND ("Randomized Controlled Trial"[pt] OR "Prospective Studies"[Mesh] OR randomized[tiab] OR randomised[tiab] OR prospective[tiab])	224	7/7: C-TRACT; Rossi; Shekarchian; VIRTUS; ABRE; VERNACULAR; VIDIO [10, 17, 74, 75, 77, 78, 79].

Domain	Exact PubMed query	Count	Selected benchmark reports retrieved
PE reperfusion	("Pulmonary Embolism"[Mesh] OR "pulmonary embolism"[tiab]) AND ("Thrombolytic Therapy"[Mesh] OR thrombolysis[tiab] OR fibrinolysis[tiab] OR "catheter-directed"[tiab] OR thrombectomy[tiab] OR embolectomy[tiab]) AND ("Randomized Controlled Trial"[pt] OR randomized[tiab] OR randomised[tiab])	493	12/12: PEITHO; HI-PEITHO; PRAGUE-26; PEERLESS; STRATIFY; ULTIMA; CANARY; SUNSET sPE; PRETHA; BETULA; STORM-PE; combined-treatment pilot [11, 12, 49, 82, 84, 85, 87, 88, 90, 91, 92, 93].
Medication after intervention	(stent[tiab] OR stenting[tiab] OR thrombectomy[tiab] OR "catheter-directed"[tiab]) AND (venous[tiab] OR vein[tiab] OR "pulmonary embolism"[tiab]) AND (aspirin[tiab] OR rivaroxaban[tiab] OR apixaban[tiab] OR dabigatran[tiab] OR edoxaban[tiab] OR warfarin[tiab] OR antithrombotic[tiab] OR antiplatelet[tiab]) AND ("Randomized Controlled Trial"[pt] OR randomized[tiab] OR randomised[tiab])	80	2/2: ARIVA; RE-SPIRE [13, 96].

Common date filter: publication date 1950/01/01–2026/09/10 (PubMed ESearch: datetype=pdat; mindate=1950/01/01; maxdate=2026/09/10). No language or species filter was added. Counts total 1154 before deduplication and 1002 unique PubMed identifiers. The 1002 records comprised 1000 journal records and two book-based evidence reports. These are retrieval counts, not counts of included studies. Benchmark reports were matched by DOI to PMID and checked against each domain result set. The benchmark is selected and known in advance; 32/32 retrieval is not an estimate of overall search sensitivity. The two-year CLOUT conference report was outside this PubMed benchmark. Guidelines were sought separately. Indexing updates can change later query counts.

The relevance check added discussion of PRAIS, OPTALYSE PE, and SPECIAL, together with the PEERLESS II design report [36, 94, 95, 109]. These were selected for their direct relevance to post-procedure medication or procedure choice. Reviews, protocols without results, and studies outside the established DVT/PE question were not treated as additional efficacy comparisons; older and overlapping reports were considered within the narrative scope. A separately identified STORM-PE 90-day report, published on 5 October 2026 and therefore outside the core publication cutoff, was incorporated during targeted verification [89]. This check does not establish that all relevant reports were included.

Targeted checks also addressed epidemiology, outcome populations, PE guideline recommendations, and the availability of PEERLESS II and PEITHO-3 results. Zhen et al.'s corrigendum adds funding information without changing the epidemiological estimates [1, 110]. The Chinese population death rate, Polish in-hospital case fatality, and US Medicare treatment-uptake estimate describe different quantities [1, 2, 3]. Results availability for the two PE trials is reported in Section S11. Except for the explicitly identified STORM-PE follow-up report, these checks did not extend the publication cutoff or update the full registry snapshot.

S3. Qualitative evidence appraisal

Extraction covered population, anatomy, disease stage, intervention, comparator, analysis sample, outcomes, follow-up, and harm. We considered masking, missing data, co-interventions, multiplicity, applicability, and sponsor involvement. Table S2 summarizes judgments without assigning a new formal risk-of-bias score or GRADE rating. Related reports from one trial were treated as overlapping evidence.

Table S2. Selected evidence and qualitative appraisal, grouped as acute DVT/compression, chronic reconstruction, PE, medication/prophylaxis, and guidance.

Study and sample	Design or source limitation	Use in the review
ATTRACT [9, 26]<br>692 randomized; 691 analyzed	Open treatment; blinded assessment; secondary efficacy p≤0.01. Iliofemoral n=391 overlaps.	Symptoms and severity; no routine any-PTS prevention.
CaVenT [41, 42]<br>209 randomized	189 assessed at two years; 176 at five. Open treatment; examiner initially unaware.	Assessed-patient PTS, with early bleeding and missingness.
CAVA [43, 44]<br>184 randomized; 152 mITT	Primary report assessed through a publication-derived thesis chapter; full long-term report; 120 assessed long term.	Definition-sensitive PTS outcomes and attrition.
IDEAL DVT [39,138]<br>865 randomized	Results abstract plus separate protocol; full results methods unavailable.	Individualized duration after six initial months of compression.

Study and sample	Design or source limitation	Use in the review
CLOUT [45]<br>500 enrolled; 499 analyzed	Indexed record and published Results section; single arm; co-interventions.	Feasibility and recovery, not comparative benefit.
CLOUT follow-up [46, 59]	One-year article; two-year presentation; assessed-limb denominators; attrition.	Descriptive durability with publication-status qualification.
CLOUT–ATTRACT matched comparison [65]<br>164 patients per group	Propensity matching across separate studies; residual confounding and differences in treatment era and outcome assessment.	Association with less PTS; no randomized estimate of thrombectomy benefit.
Historical DVT comparisons [60, 61]	Primary abstracts; older techniques and substantial co-interventions.	Historical randomized context; limited modern-device applicability.
PATH/ATTRACT interaction [15, 16]	Full reports; post hoc per-protocol analysis n=381.	Risk and treatment-effect models differ; no validated cutoff.
Puche Palao [111]<br>176 patients	Full report; observational, imbalanced groups; modified PTS definition.	Measurement and selection example.
Rossi [74]<br>51 allocated limbs	Full report; mixed lesions, correlated limbs, baseline imbalance.	Earlier randomized context; not 51 independent patients.
Shekarchian [75]<br>63 randomized	Full report; 50 complete primary QoL cases; early stopping.	Imputation supports direction; scoring scales differ.
C-TRACT [10]<br>225 randomized; 224 mITT	Full accepted manuscript; combined intervention; blinded VCSS, unblinded QoL; six months.	Treatment-package benefit with more bleeding.
ARIVA [13]<br>169 randomized; 156 efficacy	Full report; early stopping; blinded efficacy adjudication; wide interval.	Aspirin superiority unresolved, not equivalence.
VIDIO [17]<br>68 stented; 48 nonthrombotic, 20 post-thrombotic	Full report (including eligibility supplement); imaging-selected treated cohort; no untreated control.	Post-treatment prognosis, not treatment-effect selection.
ABRE/VERNACULAR [78, 79]	Full reports; single-arm cohorts; mixed disease and patency methods.	Population-specific outcomes; no device ranking.
ARIVA functional analysis [76]	Full report; post hoc; inconsistent denominators, interval, and scale wording.	Qualitative recovery only; affected estimates omitted.
PRAIS [36]<br>72 randomized; 67 analyzed	Full report; exploratory open-label trial; post-randomization omissions; sparse events.	Medication after acute DVT intervention; no equivalence conclusion.
PEITHO acute [82]<br>1006 randomized; 1005 ITT	Full report; double-blind; blinded adjudication; serious bleeding.	Early benefit–harm tradeoff.
PEITHO follow-up [83]<br>709 at selected sites	Full report; outcome-specific survivor samples; limited late adjudication.	No demonstrated durable benefit; incomplete ascertainment.
HI-PEITHO [11]<br>544 randomized; 542 safety	Full report; distress-enriched population; blinded adjudication; seven days.	Clinical composite benefit; uncertain survival and bleeding effects.
PRAGUE-26 [49]<br>558 randomized	Full report; unblinded care and adjudication; seven-day endpoint.	Early composite benefit; no survival or equal-safety claim.
PEERLESS [12]<br>599 randomized; 550 treated	Full report; post-randomization exclusions; ICU in primary hierarchy.	Procedure choice; no medical-control comparison.
STORM-PE [84]<br>100 randomized	Full acute report; blinded 48-hour imaging; sponsor operational roles. Later 90-day report assessed from the published abstract.	Early imaging efficacy; secondary functional signal with incomplete follow-up; no safety-equivalence claim [89].
STRATIFY [85, 86]<br>210 randomized	Full report; blinded imaging; 189 change assessments; bleeding discrepancy.	Separate pooled-lysis and delivery-route contrasts.

Study and sample	Design or source limitation	Use in the review
ULTIMA/SUNSET sPE [87, 90]	Primary records; SUNSET full report; different comparators.	Medical-control versus procedure-comparison questions.
CANARY [88]<br>94 randomized; 85 assessed	Full report; early cessation; imprecise primary three-month outcome.	Secondary imaging findings cannot replace the primary result.
PRETHA/BETULA/pilot [91, 92, 93]	PRETHA full report; others primary abstracts; small mechanistic trials.	Exploratory evidence, not durable clinical-effect estimates.
OPTALYSE PE [94]<br>101 randomized	Primary abstract; four lytic regimens; imaging endpoint; no medical-only group.	Dose/duration research, not an indication for reperfusion.
SPECIAL [95]<br>27 randomized	Full report; single center; early termination; imaging NI not met.	Procedure comparison; post hoc superiority is exploratory.
RE-SPIRE [96]<br>66 randomized; 63 analyzed	Full report; unmasked treatment, blinded adjudication; sparse events.	Medication after reperfusion, not reperfusion efficacy.
RENOVE/API-CAT [33, 34]	Full reports; distinct populations, NI margins, horizons, and models.	Extended treatment; conclusions remain trial-specific.
Cancer treatment/prophylaxis [101, 102, 103, 112, 113]	Hokusai/SELECT-D/AVERT full reports; Caravaggio/CASSINI publisher Methods/Results; appendices not comprehensively reviewed.	Phase-specific efficacy and bleeding comparisons (Table S5).
EPCAT III [114, 115]<br>5429 randomized; 5365 mITT	Full report and correction; double-blind NI trial; distinct safety analyses.	Regimen-specific arthroplasty prophylaxis.
Stengel [116]<br>15 studies	Full review; heterogeneous prevention regimens and ascertainment.	Perioperative stockings context only.
ICS/ESC/ISTH [27, 28, 30, 58]	Original relevant recommendation text; differing dates and grading systems.	Population- and phase-specific guidance.
Chronic venous guidance [47, 48]	ESVS 58, 61–62; SCAI 7.1; predates C-TRACT.	Selection and surveillance, not independent efficacy evidence.
ESC/ERS follow-up [97]	Full position paper; guidance, not a trial.	Functional review and symptom-triggered investigation.

Confidence intervals and analysis populations should be read with the reported endpoint. A negative superiority test does not establish equivalence, and a failed noninferiority test does not establish inferiority. Funding was considered alongside disclosed operational roles, not as proof of bias. C-TRACT had NIH sponsorship; ARIVA had academic sponsorship with industry and institutional support. Industry participated in HI-PEITHO, PEERLESS, CLOUT, and STORM-PE to differing degrees [10, 11, 12, 13, 46, 84].

S4. Analysis populations and source limitations

We distinguish limitations of a study from limitations of source access. Full main-report access does not imply access to every protocol, statistical analysis plan, or appendix. The notes below identify discrepancies that affect interpretation; uncertain values were omitted or retained explicitly as published, rather than silently corrected.

S4.1. Guidance and report versions

Relevant original guidance was consulted for ESVS, ESVM, ASH, NICE, ICS, ESC, ISTH, AVF, the International Initiative on Thrombosis and Cancer (ITAC), SCAI, and the NIVL consensus. CHEST 2021 was reviewed in the complete journal pre-proof; the SIR chronic-obstruction statement was also available as a pre-proof. The final typeset versions were not compared with the pre-proofs used. Acute SIR guidance was checked in the complete publisher report, including its appendices. Its evidence letters and recommendation strengths are distinct from ESVS classes or GRADE certainty [4, 5, 6, 7, 8, 27, 28, 29, 30, 40, 47, 48, 51, 52, 53, 54, 58, 72, 73, 100, 104].

SIR Recommendations 3 and 3c support selected CDT/PCDT, including persistent substantial symptoms, but do not impose a universal period of failed anticoagulation before intervention. Recommendation 8 generally discourages mechanical thrombectomy without lytics, while allowing selected severe presentations with lysis contraindications. Recommendation 10 concerns residual flow-limiting iliac lesions with adequate inflow. ESVS 2021 Recommendations

34–36 distinguish anatomy and continuing anticoagulant duration. PE recommendation classes were verified against the original guideline documents and are summarized in Table S7 [4, 50, 51, 53].

S4.2. Acute DVT and compression

CaVenT: we examined the two- and five-year publisher reports [41, 42]. Of 209 randomized patients (101 CDT; 108 control), 189 had two-year outcomes (90 CDT versus 99 control) and 176 had five-year outcomes (87 versus 89). PTS occurred in 37/90 versus 55/99 at two years and 37/87 versus 63/89 at five years. These are assessed-patient denominators. The reports label their analyses intention to treat, but exclude ineligible participants and unavailable outcomes under stated missingness assumptions. Five-year PTS is visit status: some patients crossed below the diagnostic threshold after meeting it at two years.

CaVenT enrolled high proximal DVT with symptoms within 21 days and excluded previous ipsilateral proximal DVT. Asymmetric venographic ascertainment prevents reconstruction of a uniform modern iliofemoral stratum. Treatment was open-label; the follow-up examiner had no prior knowledge of allocation. A published five-year missing-outcome scenario retained a 19-percentage-point reduction (95% CI 5–33), but cannot address every missingness mechanism. Severe PTS counts were four versus one, and quality-of-life benefit was not demonstrated. Three major acute bleeding events occurred with CDT. Ambiguous severe-PTS percentages and selected quality-of-life table labels were not reproduced; the supported PTS comparisons were retained [41, 42].

ATTRACT randomized 692 patients and analyzed 691. Its prespecified secondary-efficacy threshold was $p \leq 0.01$, so moderate/severe PTS at $p=0.04$ did not meet that threshold. The 391-patient iliofemoral analysis belongs to the same trial. A later exploratory, per-protocol interaction analysis included 381 patients receiving assigned treatment within seven days and does not validate an eligibility score [9, 15, 16, 26]. The propensity-matched comparison of CLOUT thrombectomy patients with patients from the ATTRACT anticoagulation arm remained a nonrandomized comparison between separate studies [65].

The primary CAVA assessment relied on its indexed record and a thesis chapter adapted from the publication; its long-term report was available in full (PMC8483549). Of 184 randomized patients, 152 entered the original modified intention-to-treat analysis and 120 were assessed long term. Original and ISTH PTS definitions produced different conclusions, emphasizing the importance of the chosen definition and follow-up population [43, 44].

The 2014 IDEAL DVT protocol [138] was read separately from the indexed 2018 results report [39]. Randomization occurred 2–6 weeks after DVT, during the common six-month compression period. The protocol allowed later discontinuation according to repeated Villalta assessments, reassessment, and restarting treatment. The 865 randomized patients and PTS incidences of 29% versus 28% come from the results report; full final-report methods were unavailable. The trial addresses duration, not compression versus no compression [39].

Puche Palao et al. used a count of at least five signs/symptoms rather than a summed Villalta score, with substantial age and cancer imbalance. The observational comparison illustrates measurement and selection limitations [111]. FILTER-PEVI was available as a primary abstract: 141 patients undergoing intervention were randomized to filter or no filter. PE testing was symptom-triggered; the reported symptomatic-subset denominators must not replace the randomized arm denominators [71].

S4.3. Chronic reconstruction and post-stent medication

The full accepted manuscript of C-TRACT (PMC13078690) provided the eligibility and bleeding data summarized below. Eligibility required DVT ≥3 months earlier, substantial limitation with VCSS ≥8, Villalta ≥10, or an open ulcer, and iliac occlusion or ≥50% stenosis. Poor common femoral inflow and previous stenting were exclusions. Recruitment targets were revised without treatment-arm-specific data, and 225 patients were randomized; 224 entered modified intention-to-treat analysis. Six-month VCSS assessment was blinded, whereas patient-reported quality of life was unblinded. The intervention combined reconstruction with enhanced antithrombotic treatment. Its VCSS difference was −2.0 (95% CI −3.2 to −0.8); a minimal clinically important difference has not been formally established [10].

Table 3 of the C-TRACT primary report counts patients: any bleeding 13/112 versus 4/112; major bleeding 4/112 versus 1/112; nonmajor bleeding 11/112 versus 3/112. Categories overlap and should not be added or derived by subtraction. The reported adjusted all-bleeding risk ratio was 3.22 (95% CI 1.07–9.69), with nominal $p=0.03$. Major bleeding in the intervention arm occurred more than 90 days after the procedure. The Discussion states that most bleeding events occurred months after intervention. This timing directs attention to continuing medication, but does not identify a causal drug component. The separate appendix was not reviewed [10].

ARIVA randomized 169 patients, with 162 in the full analysis set and 156 in the primary efficacy population (77 versus 79). Primary patency was 73/77 versus 73/79. The publisher report gives a difference of 2.4 percentage points with a 95% CI of −13.6 to 18.0; the method behind the interval's width could not be resolved from the main report, so the published interval is retained. No major bleeding occurred. Clinically relevant nonmajor bleeding affected 7/85 versus 2/84 patients; patient counts differ from event counts. Efficacy adjudication was blinded, while bleeding was investigator-reported. Early stopping and imprecision limit the negative superiority result [13].

The ARIVA post hoc functional report contains conflicting total samples (149 in the narrative; 142 in Table 2 of that functional report, whose subgroups total 149), an estimate outside its stated confidence interval, and inconsistent CIVIQ-20 direction wording. We retain the qualitative recovery description, omit affected estimates and simulated-control NNTs, and do not infer that these discrepancies invalidate the parent medication trial [76].

Rossi's trial allocated 51 qualifying limbs from 50 patients contributing 85 assessed limbs; bilateral qualifying limbs shared allocation. Within-patient correlation adjustment was not described, and the abstract/highlights differ from the main tables on cohort labels, pain, and migration. Shekarchian randomized 63 patients, with 50 complete primary quality-of-life cases. Multiple imputation supported the direction of its relative-scale estimate, but original and intrinsic VEINES-QoL scoring remain distinct [14, 74, 75].

VIDIO enrolled 100 patients and stented 68: 48 with nonthrombotic lesions and 20 with post-thrombotic disease (64 with six-month revised VCSS data). An IVUS area-stenosis threshold >54% predicted clinical improvement in the mixed treated cohort (AUC 0.64; 95% CI 0.51–0.76; p=0.04), whereas a diameter threshold >61% concerned the nonthrombotic subgroup. These associations predict improvement among selected treated patients rather than a comparative treatment effect across thresholds [17].

S4.4. CLOUT follow-up populations

The one-year CLOUT article and two-year conference presentation were examined separately. Of 500 enrolled patients, one was excluded, leaving 499 patients and 521 limbs in the full analysis population. At one year, 310 patients completed visits, but Villalta outcomes used 285 limbs: PTS 55/285 (19.3%) and moderate/severe PTS 25/285 (8.8%). At two years, 285 patients completed follow-up and Villalta outcomes were reported as percentages among 265 assessed limbs (18.5% and 6.4%, respectively, without exact integer event counts in the conference presentation). The presentation's different one-year data cut was not substituted for the peer-reviewed report. These uncontrolled, assessed-limb results do not estimate PTS incidence among all enrollees or benefit over anticoagulation [45, 46, 59].

S4.5. PE clinical-outcome trials

PEITHO's acute and long-term publisher reports were examined separately. The acute report distinguishes 1006 randomized from 1005 analyzed participants (506 versus 499). Major extracranial bleeding was 32/506 versus 6/499; ISTH major bleeding was 58/506 versus 12/499. All stroke was 12/506 versus 1/499, including hemorrhagic stroke in 10/506 versus 1/499. These categories overlap. Acute events were independently adjudicated with allocation masked; the primary effect measure is an odds ratio, not a risk ratio [82].

Long-term PEITHO assessment involved 709 participants at 28 of 76 sites. Mortality was reported for 73/359 versus 63/350 participants (20.3% versus 18.0%); the smaller vital-status ascertainment counts are not the denominators for those percentages. Symptoms were assessed in 175 versus 183 survivors and echocardiography in 144 versus 146. CTEPH was reported in 4/190 versus 6/186. Late causes of death and CTEPH were not independently adjudicated, and CTEPH investigation was triggered by symptoms or echocardiography. Selected p values in Table 4 of the primary follow-up report conflict with the displayed echocardiographic counts, so interpretation here rests on the reported counts. Incomplete ascertainment qualifies the absence of demonstrated durable benefit [83].

HI-PEITHO randomized 544 patients (273 versus 271); the treated safety groups contained 271 each. Patients were aged 18–80 years, had acute PE in a main or proximal lobar artery, RV/LV ≥1.0, elevated troponin, and at least two new distress markers within six hours before randomization. The markers were systolic pressure ≤110 mmHg for ≥15 minutes; heart rate ≥100/min, not explained by hypovolemia, arrhythmia, or sepsis; and respiratory rate >20/min or hypoxemia (room-air SpO2 <90% or arterial oxygen pressure <60 mmHg). Persistent hemodynamic instability and symptoms lasting >14 days were exclusions; these are selected criteria, not the complete eligibility list. The main report and ClinicalTrials.gov record NCT04790370 were checked. Seven-day major bleeding was 11/271 versus 6/271, with reported RR 1.8 (95% CI 0.7–4.9). Blinded adjudication supports the composite result, while sparse deaths and bleeding events limit separate inferences [11].

In HI-PEITHO, decompensation included a National Early Warning Score (NEWS) ≥9 between 24 hours and 7 days after randomization, confirmed on two measurements 15 minutes apart. This was the only qualifying decompensation criterion in 1 intervention patient and 14 controls [11].

PRAGUE-26 included 558 randomized patients (280 versus 278), all with seven-day follow-up. Selected inclusion criteria were age >18 and ≤80 years, proximal PE with symptoms <14 days, sPESI ≥1, RV/LV ≥0.9 on CT or echocardiography, and elevated high-sensitivity troponin or natriuretic peptide. It did not require HI-PEITHO's two distress markers. Eligibility was cross-checked against ClinicalTrials.gov NCT05493163 on 10 September 2026; the full bleeding-risk and other exclusions must also be applied. Its eligibility and decompensation definitions differed from HI-PEITHO, and the clinical events committee knew allocation. Seven-day ISTH major bleeding was 5/280 versus 7/278, GUSTO major bleeding 4/280 versus 6/278, and intracranial hemorrhage 2/280 versus 0/278. Secondary intervals were not multiplicity-adjusted. The endpoint could include a sustained NEWS ≥9; clinician escalation could

also occur without meeting the endpoint. The separate appendix was unavailable, so the effect was not attributed to a particular decompensation component. Thirty-day follow-up was less complete in controls [49].

PEERLESS randomized 599 patients but enrolled/treated 550 after intraprocedural eligibility assessment. The 49 exclusions included 35 meeting the prespecified pulmonary artery pressure exclusion. Its primary hierarchy assessed death, intracranial hemorrhage, major bleeding, deterioration or bailout, and ICU admission/length of stay through discharge or seven days, whichever occurred first. Major bleeding affected 19 patients in each treatment group (6.9% each), and the secondary four-component hierarchy excluding ICU stay was inconclusive. Because the two win ratios reflect different hierarchical endpoint definitions, their numerical difference does not isolate the independent effect of ICU admission. There was no anticoagulation-only group [12].

S4.6. Imaging-focused PE trials

STORM-PE randomized 100 patients (47 versus 53). The primary RV/LV analysis used 46/47 and 52/53 assessments, assigning zero change after rescue without reassessment. Seven-day major adverse events affected 2/47 versus 4/53, PE-related deaths two versus zero, and major bleeding one versus one. Components overlap. Adjudication as unrelated to the device or procedure does not remove deaths from the randomized comparison. Imaging was assessed blinded; the industry sponsor had operational roles. The 48-hour between-group difference of 0.27 reflects the published estimate alongside the rounded group means of 0.52 and 0.24 [84].

The separate STORM-PE 90-day report was published on 5 October and assessed from the published abstract on 14 October 2026. Six-minute walk testing was completed by 31/47 thrombectomy patients and 34/53 controls. Mean distances were 478.7 ± 159.5 versus 368.0 ± 127.8 m (reported $p=0.003$); NYHA class I was recorded in 37/38 versus 34/45 assessed participants. Dyspnea and quality-of-life measures did not show corresponding between-group differences. The findings suggest a functional recovery signal among assessed patients. Incomplete follow-up and the small sample limit conclusions about durability, survival, and comparative safety [89].

STRATIFY allocated 71, 70, and 69 patients to ultrasound-assisted treatment, intravenous alteplase, and heparin. Primary clot-score change was available for 189 patients. The two lytic groups were pooled for one contrast and compared with each other for another. Overall bleeding counts differ between the narrative and Table 2 of the STRATIFY primary report, so no pooled count was adopted. TIMI major bleeding (2/71, 2/70, 0/69) and three-month deaths (2/71, 4/70, 0/69) retain their own definitions and denominators [85, 86].

PRETHA had 13 patients per arm, mechanistic primary endpoints, and baseline functional imbalance. CANARY randomized 94 patients, with 85 assessed for its three-month primary outcome after early stopping. BETULA and the combined-treatment pilot were assessed from their published abstracts. These studies add randomized evidence but remain too imprecise for firm mortality, durable-function, or safety-equivalence claims [88, 91, 92, 93].

OPTALYSE PE randomized 101 patients to four ultrasound-facilitated lytic dose/duration regimens. The primary abstract reports RV/LV improvement within each treatment group and records major bleeding and intracranial hemorrhage [94]. The full SPECIAL report describes 27 patients randomized to ultrasound-assisted lysis or surgical embolectomy. The trial stopped early and its 72-hour imaging comparison was inconclusive for noninferiority. A post hoc superiority analysis favored surgery [95]. Both studies address technique choice among intervention candidates. Imaging endpoints, limited samples, and the absence of a medical-only group constrain their implications for treatment selection and clinical safety.

S4.7. Medication and prophylaxis comparisons

RENOVE and API-CAT were assessed in their main reports. RENOVE was open-label with blinded adjudication; API-CAT used double-blind treatment and central blinded adjudication. RENOVE's failed primary noninferiority test blocked confirmatory secondary testing, despite a favorable bleeding estimate. Its five-year Kaplan–Meier estimates differ from API-CAT's 12-month competing-risk cumulative incidences. Population, endpoints, margins, and analysis models also differ, precluding a direct cross-trial drug comparison [33, 34].

EPCAT III randomized 5429 participants; 5365 entered modified intention-to-treat analysis (2718 versus 2647), leaving 64 randomized participants outside that analysis. At-least-one-dose safety populations were 2681 versus 2600. Table S5 keeps these analyses separate. Noninferiority concerned the primary proximal-DVT/PE endpoint; the distal-DVT extension was post hoc. Long-term aspirin users (431/5365) continued low-dose open-label aspirin in addition to their assigned regimen. The correction clarifies distal-DVT wording without changing the numerical results. Double-blind EPCAT III and unblinded cluster-crossover CRISTAL cannot be compared as if masking alone explained their different findings [114, 115, 117].

RE-SPIRE randomized 66 patients after reperfusion and analyzed 63 (32 versus 31) on treatment. Recurrent VTE was 1/32 versus 1/31, major bleeding 0/32 versus 1/31, and reported minor bleeding 0/32 versus 5/31. The publication and registry indicate unmasked treatment with blinded central adjudication. Reasons for three post-randomization omissions and selected p values differ within the report; the review therefore uses the counts without superiority or noninferiority claims. Its old registry target is not evidence of current recruitment [96].

PRAIS was an exploratory, open-label, multicenter comparison after successful treatment of acute iliofemoral DVT. Of 72 randomized patients, 67 were included in the reported analysis: 35 receiving rivaroxaban and 32 enoxaparin/warfarin. Six-month recurrent VTE occurred in 4/35 versus 4/32, and major or clinically relevant nonmajor bleeding in 1/35 versus 3/32. These exploratory estimates inform medication choice after intervention, while the small sample and post-randomization omissions leave comparative efficacy and safety uncertain [36].

Stengel et al.'s perioperative stockings review combines heterogeneous prophylactic regimens and ascertainment methods. Its pooled estimate was not presented as the effect of adding stockings to anticoagulation, or extrapolated to intermittent pneumatic compression or symptom treatment after established DVT [116].

S5. Selected historical milestones

Table S3. Selected historical milestones.

Period / source	Development	Change in therapeutic objective	Historical boundary
1916 [18]	McLean reports anticoagulant observations during cephalin research.	Experimental foundation for pharmacological control of coagulation.	Laboratory discovery was not evidence of clinical VTE efficacy; later purification and translation were essential.
Oral anticoagulant development; historical account 1959 [19]	Dicumarol and subsequent coumarin development described by Link.	An oral route for sustained anticoagulation.	Historical discovery and a modern randomized efficacy comparison are different evidence types.
1960 [20]	Barritt–Jordan controlled study in patients presenting with suspected PE.	Prevention of recurrent and fatal embolism with anticoagulation.	This did not study primary prophylaxis in patients without PE. Historical diagnosis and trial methods differ from current standards.
1996 [21, 22]	Randomized home-based LMWH versus inpatient UFH strategies.	Safe ambulatory management becomes feasible for selected proximal-DVT patients.	Suitability for outpatient care remains a clinical selection decision.
1997 ten-year report [62]	Open thrombectomy, temporary AV fistula, and anticoagulation.	Long-term venous patency and post-thrombotic morbidity become explicit goals.	Only 30 patients returned at ten years; not a modern device comparison.
2010–2013 [23, 24]	Rivaroxaban and apixaban VTE trials.	Simpler oral treatment without making thrombus extraction a prerequisite.	Drug-specific regimens and trial populations must be retained.
2012–2020 [9, 41, 43]	CaVenT, ATTRACT, and CAVA test adjunctive catheter thrombolysis.	Formal evaluation of PTS prevention, severity, and clinical trade-offs.	Heterogeneous findings and bleeding preclude a universal clot-removal mandate.
2025–2026 [10, 11, 13]	ARIVA, C-TRACT, and HI-PEITHO distinguish post-stent therapy, chronic reconstruction, and pulmonary reperfusion.	Benefit is evaluated against a decision-specific comparator.	No single trial resolves all stages or venous territories.

Dates identify selected publications or developments, not claims of historical priority. AV, arteriovenous; LMWH, low-molecular-weight heparin; UFH, unfractionated heparin.

S6. Dedicated-stent cohorts

Table S4. Dedicated venous stent cohorts and estimation methods.

Study / population	12-month primary patency	Longer follow-up	Interpretive boundary
VIRTUS pivotal: 170; 127 post-thrombotic [77]. Later report combines 170 pivotal + 30 feasibility patients [80].	Overall 84.0%; nonthrombotic 96.2%; post-thrombotic 79.8%. Primary endpoint based on venographic patency.	36 months: 86/120 (71.7%) overall; 27/28 (96.4%) nonthrombotic; 59/92 (64.1%) post-thrombotic.	Later denominator and pooled cohorts differ from pivotal enrollment. Complete-case proportions are not interchangeable with Kaplan–Meier estimates.
ABRE: 200; acute DVT 33, PTS 95, NIVL 72 [78, 81].	Overall 162/184 (88.0%); acute DVT 27/31 (87.1%); PTS 67/84 (79.8%); NIVL 68/69 (98.6%).	36-month Kaplan–Meier estimates: overall 81.6%; acute DVT 76.5%; PTS 70.4%; NIVL 97.1%.	Acute-DVT cohort involved residual obstruction after thrombus removal. Study definition includes freedom from occlusion, ≥50% restenosis, and clinically driven revascularization.
VERNACULAR: 170; PTS 93, NIVL 77 [79].	Weighted overall estimate 88.6%; Results section subgroup estimates: PTS 81.3%, NIVL 96.9%.	36-month binary estimate 79.5% overall (PTS 70.0%, NIVL 93.6%); Kaplan–Meier overall estimate 84.0%. 128 completed 3-year follow-up.	Primary patency: freedom from TVR, thrombotic occlusion, or >50% stenosis. The weighted 12-month, binary 36-month, and KM estimates are different statistics.

Single-arm cohorts describe outcomes in selected populations. Disease mix, patency definition, follow-up completeness, and binary versus Kaplan–Meier estimation prevent device ranking. VERNACULAR's Results-section subgroup estimate is used where its Discussion gives a different rounded value. PTS, post-thrombotic syndrome; NIVL, nonthrombotic iliac vein lesion; TVR, target vessel revascularization; KM, Kaplan–Meier.

S7. Selected anticoagulation and prophylaxis trials

Table S5. Anticoagulation and prophylaxis trials.

Trial and comparison	Population and horizon	Efficacy outcome	Bleeding and interpretive boundary
RENOVE [33] Reduced versus full-dose apixaban/rivaroxaban	Open-label; independent blinded adjudication. 2768 randomized; extended treatment after 6–24 months full dose. Median follow-up 37.1 months; estimates are 5-year cumulative incidences.	Recurrent VTE 2.2% versus 1.8%; adjusted HR 1.32 (95% CI 0.67–2.60). Noninferiority not met. NI margin HR 1.70; failure is not demonstrated inferiority.	Major or clinically relevant nonmajor bleeding 9.9% versus 15.2%; adjusted HR 0.61 (0.48–0.79). Failed efficacy NI blocked confirmatory secondary testing; the favorable bleeding estimate does not establish equal efficacy.
API-CAT [34] Reduced versus full-dose apixaban	Double-blind; blinded central adjudication. 1766 ITT (866 versus 900); active cancer, ≥6 months prior treatment without symptomatic recurrence; 12-month horizon.	Cumulative VTE 2.1% versus 2.8%; adjusted subdistribution HR 0.76 (0.41–1.41); NI margin 2.00, p=0.001.	Clinically relevant bleeding 12.1% versus 15.6%; adjusted subdistribution HR 0.75 (0.58–0.97), p=0.03 within hierarchy. Major bleeding 2.9% versus 4.3%; 0.66 (0.40–1.10), nonconfirmatory component. Competing-risk estimates are not crude percentages.
Caravaggio [101] Apixaban versus dalteparin	Cancer-associated acute VTE; 1170 randomized, 1155 modified ITT (576 vs 579); 6 months.	Recurrent VTE 5.6% versus 7.9%; HR 0.63 (0.37–1.07); noninferiority met.	Major bleeding 3.8% versus 4.0%; HR 0.82 (0.40–1.69). Nonsignificance is not proof of identical bleeding risk.

Trial and comparison	Population and horizon	Efficacy outcome	Bleeding and interpretive boundary
Hokusai VTE Cancer [102]<br>Edoxaban after ≥5 days LMWH versus dalteparin	1050 randomized; 1046 modified ITT. Treatment 6–12 months; endpoint horizon 12 months regardless of duration.	Primary recurrent-VTE/major-bleeding composite 12.8% versus 13.5%; HR 0.97 (0.70–1.36), noninferiority met. Recurrent VTE 7.9% versus 11.3%.	Major bleeding 6.9% versus 4.0%; risk difference 2.9 pp (0.1–5.6). Composite noninferiority does not imply equality of its components.
SELECT-D [103]<br>Rivaroxaban versus dalteparin	406 randomized; open-label pilot. Six-month cumulative estimates.	Recurrent VTE 4% versus 11%; HR 0.43 (0.19–0.99).	Major bleeding 6% versus 4%; HR 1.83 (0.68–4.96). CRNMB 13% versus 4%; HR 3.76 (1.63–8.69). Pilot trial; retain imprecision.
AVERT [112]<br>Apixaban 2.5 mg twice daily versus placebo	Khorana ≥2 starting chemotherapy; 574 randomized, 563 modified ITT; 180 days.	VTE 4.2% versus 10.2%; HR 0.41 (0.26–0.65).	Major bleeding in modified ITT 3.5% versus 1.8%; HR 2.00 (1.01–3.95). Treatment-period bleeding estimates are different and are not substituted here.
CASSINI [113]<br>Rivaroxaban 10 mg daily versus placebo	Khorana ≥2; no DVT on baseline screening; 841 randomized; 180 days. Serial screening contributed events.	Primary composite 6.0% versus 8.8%; HR 0.66 (0.40–1.09). Intervention-period analysis 2.6% versus 6.4%; HR 0.40 (0.20–0.80).	Major bleeding 2.0% versus 1.0% in safety populations (405 versus 404); HR 1.96 (0.59–6.49). The supportive intervention-period result does not replace the nonsignificant primary analysis.
EPCAT III [114]<br>Aspirin versus rivaroxaban for 5 days, then aspirin	Double-blind; independent adjudication. 5429 randomized; 5365 mITT (2718 versus 2647); elective hip/knee arthroplasty; 90 days.	Proximal DVT/PE 0.48% versus 0.45%; RD 0.02 pp (−0.34 to 0.39); NI margin 0.7 pp. Post hoc with distal DVT: 1.03% versus 0.87%; RD 0.16 pp (−0.36 to 0.68).	mITT major/clinically relevant nonmajor bleeding: 45/2718 (1.66%) versus 54/2647 (2.04%); RD −0.38 pp (−1.11 to 0.34). Major: 0.44% versus 0.30%; RD 0.14 pp (−0.19 to 0.46). Nonmajor: 1.21% versus 1.74%; RD −0.52 pp (−1.17 to 0.12). Separate ≥1-dose safety analysis: 40/2681 (1.49%) versus 52/2600 (2.00%); RD −0.51 pp (−1.21 to 0.21). No safety equivalence or second confirmatory NI test.

Pairs are first-listed treatment versus comparator; intervals are 95% confidence intervals. We examined the full main reports of RENOVE, API-CAT, EPCAT III, Hokusai VTE Cancer, SELECT-D, and AVERT, and the publisher Methods and Results sections of Caravaggio and CASSINI. Supplementary appendices were not comprehensively reviewed. Caravaggio distinguishes 1170 randomized from 1155 modified-ITT participants; CASSINI uses 841 for efficacy and 809 treated patients for safety. CASSINI includes screening-detected events. HR, hazard ratio; ITT, intention to treat; mITT, modified ITT; NI, noninferiority; pp, percentage points; CRNMB, clinically relevant nonmajor bleeding; RD, risk difference. These trials concern different phases and populations.

S8. Background: primary surgical and medical thromboprophylaxis

Primary prophylaxis addresses patients without established VTE. It is retained here to distinguish preventive regimens from treatment-dose anticoagulation and care after intervention. Procedure, mobility, cancer, previous VTE, renal function, bleeding risk, and neuraxial procedures determine the preventive plan [118, 119]. Extended prophylaxis after major abdominal/pelvic cancer surgery has randomized support, while cranial surgery requires particular attention to bleeding [100, 120, 121].

EPCAT II tested aspirin after initial rivaroxaban; CRISTAL compared aspirin monotherapy with enoxaparin; EPCAT III tested aspirin from the first postoperative night/day against rivaroxaban followed by aspirin. Their regimens and endpoints are not interchangeable [114, 115, 117, 122]. CLOTS 3, AVERT, and CASSINI address other preventive settings [112, 113, 123].

Table S6. Primary surgical and medical thromboprophylaxis: selected settings and regimen considerations.

Clinical setting	Core approach	Duration or timing	Important boundary
Major general surgery	Risk assessment; LMWH or low-dose UFH when appropriate; IPC for patients with high bleeding risk [118, 119].	Start according to operative hemostasis, VTE risk, and local protocol.	Prophylactic doses differ from treatment of established DVT; reassess when bleeding risk changes.
Major abdominal/pelvic cancer surgery	Pharmacological prophylaxis unless contraindicated; consider mechanical methods as an adjunct [100, 120].	Extended prophylaxis for approximately 4 weeks in eligible patients without high bleeding risk.	Early trial endpoints included venographic thrombosis; apply to the relevant surgical population.
Major cranial surgery	Mechanical prophylaxis; individualize addition of LMWH [118, 121].	NICE: consider LMWH 24–48 hours postoperatively when VTE risk outweighs bleeding risk.	ASH discourages routine pharmacological prophylaxis across all major neurosurgery; selected higher-risk cases differ.
Elective hip/knee arthroplasty	Anticoagulant or selected aspirin strategy; specify the initial and extended regimen [114, 117, 118, 122].	EPCAT III: 5 days aspirin or rivaroxaban, then aspirin for 9 more days (knee) or 30 (hip).	EPCAT III: predominantly standard-risk elective patients; NI for proximal DVT/PE versus sequential rivaroxaban–aspirin. No bleeding advantage or equivalent safety established. CRISTAL used full-course enoxaparin; endpoint, population, and design differences limit comparison.
Hip or proximal femoral fragility fracture	LMWH or appropriate heparin-based prophylaxis; IPC if pharmacological treatment is contraindicated [118, 121].	NICE recommends a month when VTE risk outweighs bleeding risk.	Do not assume evidence from elective arthroplasty transfers to frail fracture patients.
Major trauma	Pharmacological prophylaxis when bleeding risk permits; mechanical measures while bleeding risk is high [118].	Reassess dynamically with injury stability, procedures, mobility, and hemostasis.	A general trauma recommendation does not determine timing for every brain, spinal, or solid-organ injury.

Selected guidance, not a universal prescription. IPC, intermittent pneumatic compression; LMWH, low-molecular-weight heparin; UFH, unfractionated heparin; ASH, American Society of Hematology; NICE, National Institute for Health and Care Excellence.

S9. Guideline comparison

Table S7 complements main-text Table 1. Grades retain each society's definitions and should not be converted into a common scale. PE recommendations distinguish routine treatment in stable patients from rescue after deterioration. The AHA/ACC categories are not exact equivalents of ESC risk strata, and both ESC 2019 and AHA/ACC 2026 guidance preceded publication of HI-PEITHO and PRAGUE-26.

Table S7. Selected guideline recommendations and their original grades.

Document and scope	Anticoagulation and selection	Intervention guidance	Interpretive boundary
ASH 2020; DVT and PE [5]	DOACs preferred for most eligible adults; primary treatment 3–6 months.	Anticoagulation alone for most proximal DVT; selected symptomatic iliac/common femoral disease or limb threat may justify lysis.	Routine stockings are discouraged solely for PTS prevention; symptom relief is a separate goal.
CHEST 2021; 2024 compendium [6, 29]	Treatment phase generally 3 months; extended treatment depends on recurrence and bleeding risk.	Anticoagulation alone is suggested over thrombolytic, mechanical, or pharmacomechanical intervention (Recommendation 6; weak, moderate certainty).	Lower-risk isolated distal DVT: serial imaging for 2 weeks (Recommendations 1–2); the compendium consolidates earlier guidance.

Document and scope	Anticoagulation and selection	Intervention guidance	Interpretive boundary
ESVS 2021 [4]	Continue anticoagulation after removal for at least the otherwise indicated duration (Recommendation 36).	Selected symptomatic iliofemoral DVT: consider removal (Recommendation 34, IIa A); isolated femoral/popliteal/calf disease: no early removal (35, III B).	Early compression for pain/edema (31, I A); consider stockings for PTS prevention (32, IIa A).
NICE NG158; Recommendation 1.6.1 [52]	CDT consideration requires symptomatic iliofemoral DVT, symptoms <14 days, good functional status, expected survival ≥1 year, and low bleeding risk.	Selective CDT recommendation, retained from 2012 in the current guideline.	These selection criteria do not establish a universal threshold for MT, chronic reconstruction, or PE rescue.
ESC Working Groups; DVT consensus, online 2021 / issue 2022 [58]	High-risk distal DVT: anticoagulate; selected low-risk patients may undergo ultrasound surveillance.	Catheter-directed treatment only in selected cases; primary acute-DVT stenting or mechanical removal alone not recommended.	Predates RIDTS; consensus wording is retained and not converted into a new evidence grade.
ICS 2024; selected treatment recommendations [30]	Symptomatic calf DVT: 3 months unless contraindicated. Extended reduced-dose apixaban/rivaroxaban considered; high-risk exceptions remain.	These anticoagulant recommendations do not establish a thrombus-removal indication.	Native evidence/strength labels are not GRADE equivalents. Extended-dose advice predates RENOVE and API-CAT.
ESVM 2025; Interventional VTE [54]	Severe symptomatic iliofemoral/iliocaval acute DVT: consider catheter treatment (IIa A). Continue full-dose peri-interventional anticoagulation (I B).	Within endovascular treatment, prefer MT over CDT/USAT (IIa B); consider IVUS (IIa B). Dedicated stent if stenting is needed (I C).	A preference between techniques does not establish MT superiority over anticoagulation. Routine IVC filters are not recommended (III A).
SIR 2023; Acute iliofemoral DVT [53]	Prompt therapeutic anticoagulation remains essential (Recommendation 2: A/Strong).	Selected CDT/PCDT: Recommendation 3, B/Moderate. Against routine CDT/PCDT for isolated femoral/popliteal/tibial DVT, mild symptoms, or moderate/high bleeding risk: Recommendation 4, B/Strong.	MT without lytics: generally not recommended (Recommendation 8: D/Weak); selected severe presentations with lysis contraindications. Letters denote SIR evidence levels, words recommendation strengths.
AVF 2026; Upper-extremity DVT [72]	Oral factor Xa inhibitors preferred over VKA for at least 3 months in suitable patients.	May consider early removal for extensive disease <21 days, severe symptoms, and life expectancy >1 year.	Thoracic outlet disease requires anatomical assessment and consideration of decompression.
ESC/ERS 2019; stable PE [50]	Anticoagulation and monitoring; routine primary systemic lysis discouraged (III B).	After hemodynamic deterioration: rescue lysis I B; surgical or catheter alternative IIa C.	Rescue grades require deterioration on anticoagulation; they are not routine catheter-treatment recommendations.
ESC/ERS 2019; high-risk PE [50]	Urgent anticoagulation and reperfusion assessment.	Systemic lysis I B. If lysis contraindicated/failed: surgery I C; catheter treatment IIa C.	Catheter/surgical treatment requires appropriate local expertise and resources.
AHA/ACC multisociety 2026; categories C2–C3 [51]	Categories retain their original definitions.	CDT or MT versus anticoagulation: benefit unclear (2b C-LD).	Predates HI-PEITHO and PRAGUE-26; not an automatic eligibility rule.
AHA/ACC multisociety 2026; categories D1–D2 [51]	Advanced treatment in selected patients at risk of further deterioration.	CDT or MT may be considered (2b B-NR); systemic lysis 2b C-LD.	Decision depends on trajectory, bleeding risk, and expertise.

Document and scope	Anticoagulation and selection	Intervention guidance	Interpretive boundary
AHA/ACC multisociety 2026; category E1 [51]	Advanced treatment for cardiopulmonary failure.	Systemic lysis or CDT: 2a C-LD. MT or surgery: 2a B-NR.	Category E2 has different recommendations; do not extend E1 grades to all arrest presentations.
ESVS 2022; chronic iliac obstruction [47]	Severe symptoms/signs: consider endovascular treatment first (58, IIa B).	Without severe symptoms: neither endovascular nor surgical intervention (61, III C).	After reconstruction: duplex at day 1, 2 weeks, and regularly thereafter (62, I C). Predates C-TRACT.
SCAI 2025; chronic iliocaval obstruction [48]	Clinical and anatomical selection and technical expertise are essential.	Venoplasty/stenting plus conservative care is suggested over conservative care alone (7.1; conditional, low certainty).	Predates C-TRACT; apply to symptomatic chronic obstruction, with shared decision making.

COR, class of recommendation; LOE, level of evidence; B-NR, nonrandomized evidence; C-LD, limited data. Roman-number classes are used for ESC/ESVS and Arabic-number COR for AHA/ACC. SIR letters denote evidence levels and words denote recommendation strength. Selected recommendations do not replace the complete guideline. CDT, catheter-directed thrombolysis; MT, mechanical thrombectomy; PCDT, pharmacomechanical CDT; IVUS, intravascular ultrasound; DOAC, direct oral anticoagulant; VKA, vitamin K antagonist; IVC, inferior vena cava. Other society abbreviations are expanded in the main manuscript; ERS, European Respiratory Society; ITAC, International Initiative on Thrombosis and Cancer; AVF, American Venous Forum; ICS, International Consensus Statement; GRADE, Grading of Recommendations Assessment, Development and Evaluation.

S10. Interventional techniques

Technique selection follows the decision to intervene. Device names identify studied mechanisms and do not establish comparative rankings or current regulatory indications.

Table S8. Interventional mechanisms and relevant evidence.

Treatment class	Mechanism or example	Principal concerns	Evidence boundary
Infusion CDT	Local fibrinolytic delivery	Major bleeding, treatment duration, access complications	Randomized evidence includes CaVenT; technical recanalization does not ensure less disability [41].
Ultrasound-assisted CDT	Ultrasound-facilitated local fibrinolysis	Retains lytic-related bleeding risk	CAVA did not show a statistically significant reduction in its one-year primary PTS endpoint [43].
Rheolytic or pharmacomechanical treatment	Mechanical disruption/aspiration with variable use of a fibrinolytic; AngioJet	Hemolysis, kidney injury, bleeding, embolization	ATTRACT tested a pharmacomechanical strategy; observational kidney injury comparisons do not establish causality [9, 63].
Mechanical extraction without mandatory lysis	ClotTriever-based extraction	Large venous access, vessel injury, embolization, residual or recurrent thrombosis	CLOUT is single-arm; DEFIANCE is designed to test added benefit versus anticoagulation [45, 66].
Aspiration-based approaches	Catheter aspiration, sometimes combined with local lysis	Blood loss, access injury, embolization; risk depends on technique	Procedure-versus-procedure comparisons, including SWAM versus AngioJet, do not establish superiority over anticoagulation [64].
Venous stenting	Reconstruction of a clinically important fixed outflow lesion	In-stent thrombosis, inadequate inflow, migration, reintervention, antithrombotic-associated bleeding	Reconstructive strategy and post-stent medication require separate evaluation; neither is established by clot clearance alone [10, 13, 73].
Open venous thrombectomy	Direct extraction, historically with a temporary arteriovenous fistula	Operative morbidity, recurrent thrombosis, fistula-related management	Selected rescue role; the small historical randomized follow-up does not compare surgery with modern MT [4, 62].

Mechanical treatment may require adjunctive drugs or stenting. SWAM, Synchronized Wave Aspiration and Maceration; USAT, ultrasound-assisted thrombolysis. Its comparison with AngioJet used a venographic primary endpoint.

S11. Trial registry snapshot

Table S9 presents the seven-trial snapshot obtained on 13 September 2026. Dates within the table are the registry's Last Update Posted field. None of those records had posted results at retrieval. Estimated enrollment and completion dates do not indicate completed observations or a publication date.

Table S9. Selected trial programs on 13 September 2026.

Trial and identifier	Clinical question	Status and last update	Interpretation
DEFIANCE; NCT05701917 [67]	ClotTriever-based treatment versus anticoagulation for symptomatic iliofemoral DVT; hierarchical clinical outcome.	Recruiting; 9 September 2026	Estimated enrollment 300; estimated primary completion January 2028. No completed randomized result is implied.
PEERLESS II; NCT06055920 [105]	Large-bore thrombectomy versus anticoagulation; hierarchical mortality, deterioration, readmission, bailout, and dyspnea outcomes.	Active, not recruiting; 11 September 2026	Primary completion 30 July 2026 (actual); enrollment 1200 remains estimated. No results posted in the retrieved record.
PE-TRACT; NCT05591118 [106]	Catheter-directed therapy + anticoagulation versus anticoagulation; functional/exercise outcomes.	Recruiting; 18 May 2026	Estimated n = 500; estimated primary completion 29 March 2027. No results posted in the retrieved record.
PEITHO-3; NCT04430569 [108]	Reduced-dose intravenous alteplase versus placebo, with anticoagulation; 30-day death, hemodynamic decompensation, or recurrent PE.	Active, not recruiting; 1 July 2026	Estimated enrollment 800; primary completion 13 April 2026 (actual). No results posted; placebo-controlled masked comparison.
ASTER; NCT05171049 [124]	Abelacimab versus apixaban for cancer-associated VTE.	Terminated; 23 February 2026	Registry reason: sponsor decision. This status cannot support a claim of efficacy; no numerical treatment effect is inferred.
MAGNOLIA; NCT05171075 [125]	Abelacimab versus dalteparin in gastrointestinal/genitourinary cancer-associated VTE.	Terminated; 17 April 2026	Registry reason: sponsor decision. The comparator is dalteparin; do not infer a common cause or treatment effect for both trials.
ROXI-CAT-II; NCT07493304 [126]	Cenvacibart (REGN7508) versus apixaban for cancer-associated VTE; phase 3 program.	Recruiting; 19 August 2026	Estimated enrollment 1600; estimated primary completion in 2031. Registry status can change.

The registry describes PEITHO-3 as a masked comparison using alteplase 0.6 mg/kg (maximum 50 mg) over 15 minutes, which differs from STRATIFY's 20 mg over six hours. Completion, registry results, conference reporting, and journal publication are separate observations. Registry status fields refer to the snapshot described above. The PEERLESS II and PEITHO-3 records were subsequently rechecked for posted results on 14 October 2026; none were identified in those records [105, 108]. These targeted checks did not update the other five registry entries or change the publication cutoff. No primary results report was identified in the targeted publication checks described in Section S2; an unlocated conference presentation cannot be excluded.

S12. Additional clinical context

S12.1. Emerging anticoagulants

Factor XI/XIa inhibition has proof-of-concept evidence from knee-arthroplasty trials of an antisense oligonucleotide, abelacimab, and milvexian [127, 128, 129]. Venography-based prophylaxis endpoints do not establish efficacy for acute VTE treatment. Atrial-fibrillation findings also differ by agent: AZALEA-TIMI 71 found less clinically relevant bleeding with abelacimab, whereas OCEANIC-AF found more stroke/systemic embolism with asundexian despite less major bleeding [130, 131]. Favorable bleeding alone cannot establish efficacy for VTE.

On 13 September 2026, ASTER and MAGNOLIA were listed as terminated for sponsor decisions, while ROXI-CAT-II remained recruiting. These statuses do not establish treatment effects or a shared reason for termination [124, 125, 126].

S12.2. Paradoxical embolism and nonthrombotic compression

CLOSE, extended RESPECT, and attribution analyses support PFO closure after selected otherwise unexplained ischemic stroke when the shunt is plausibly causal [132, 133, 134, 135, 136]. They do not establish routine closure for incidental PFO with DVT or universal added benefit alongside lifelong anticoagulation.

Nonthrombotic iliac, thoracic-outlet, and renal-vein compression require syndrome-specific assessment. Asymptomatic renal-vein compression is not nutcracker syndrome. ESVS guidance favors conservative care for mild symptoms and considers reconstruction for severe persistent symptoms, with migration risk relevant to selected stenting [137]. Anatomy alone does not establish an indication for anticoagulation or thrombectomy.
